# Fourteen-Membered Macrocyclic Cobalt Complex Structure as a Potential Basis for Durable and Active Non-platinum Group Metal Catalysts for Oxygen Reduction and Hydrogen Evolution Reactions

**DOI:** 10.1021/jacs.5c01306

**Published:** 2025-04-25

**Authors:** Zhiqing Feng, Junya Ohyama, Soutaro Honda, Yasushi Iwata, Keisuke Awaya, Masato Machida, Masayuki Tsushida, Ryota Goto, Takeo Ichihara, Makoto Moriya, Yuta Nabae

**Affiliations:** † Graduate School of Science and Technology, Kumamoto University, 2-39-1 Kurokami, Chuo-ku, Kumamoto 860-8555, Japan; ‡ Faculty of Advanced Science and Technology, Kumamoto University, 2-39-1 Kurokami, Chuo-ku, Kumamoto 860-8555, Japan; § Institute of Industrial Nanomaterials (IINa), Kumamoto University, 2-39-1 Kurokami, Chuo-ku, Kumamoto 860-8555, Japan; ∥ Technical Division, Kumamoto University, 2-39-1 Kurokami, Chuo-ku, Kumamoto 860-8555, Japan; ⊥ Corporate R&D, Asahi Kasei Corporation, 2767-11 Niihama, Shionasu, Kojima, Kurashiki, Okayama 711-8510, Japan; # College of Science, Academic Institute, Shizuoka University, 836 Ohya, Suruga-ku, Shizuoka 422-8529, Japan; □ Research Institute of Green Science and Technology, Shizuoka University, 836 Ohya, Suruga-ku, Shizuoka 422-8529, Japan; △ Department of Materials Science and Engineering, Institute of Science Tokyo, 2-12-1 S8-26, Ookayama, Meguro-ku, Tokyo 152-8552, Japan

## Abstract

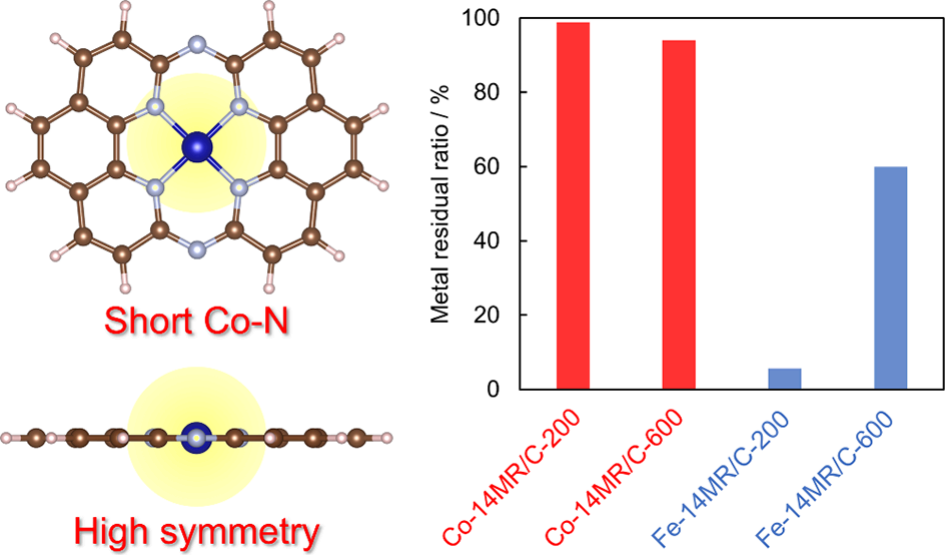

Non-platinum group metal catalysts for the oxygen reduction reaction (ORR) and hydrogen evolution reaction (HER) under acidic conditions were developed using a CoN_4_ complex with a 14-membered-ring hexaazamacrocyclic ligand (Co-14MR). The carbon-supported Co-14MR catalyst (Co-14MR/C) showed higher ORR and HER activities than a conventional carbon-supported 16-membered-ring Co phthalocyanine (CoPc/C) catalyst. Heat treatment of Co-14MR/C at 600 °C further enhanced its ORR and HER activity through structural modification of the Co active center via deprotonation of ligand amine groups. Density functional theory calculations indicated that the structural modifications of Co-14MR induced by heat treatment adjusted the adsorption energies of important intermediates in the ORR and HER toward optimal values, resulting in enhanced catalytic activity. The Co-14MR/C catalysts also exhibited higher durability in the ORR and HER than CoPc/C and Fe-14MR/C catalysts. Structural analysis suggested that the short Co–N bond lengths and small distortion of the CoN_4_ active site of the Co-14MR catalysts are the reasons for their high durability. These findings suggest that the Co-14MR structure is a promising design for non-platinum group metal catalysts for proton-exchange membrane fuel cells and water splitting.

## Introduction

The MN_4_ structure, where M = metal and N_4_ = chelating nitrogen ligand, is a promising design of non-platinum group metal (non-PGM) catalysts for electrochemical reactions for fuel cells and water splitting. In particular, FeN_4_ structures incorporated into carbon matrixes and macrocyclic complexes have attracted attention because of their relatively high oxygen reduction reaction (ORR) activity.^1−9^ However, FeN_4_ catalysts show poor stability under acidic conditions and in proton-exchange membrane fuel cells.^10,11^ Compared with FeN_4_ catalysts, CoN_4_ catalysts exhibit higher stability but lower activity for the ORR.^12−18^ The ORR activity of CoN_4_ catalysts has recently been improved by controlling the local structure of CoN_4_ active sites embedded in carbon.^19−21^ These studies raised the ORR activity of CoN_4_ catalysts while retaining durability by tuning CoN_4_ active sites. However, CoN_4_ catalysts still fall short of achieving activity levels comparable to those of FeN_4_ catalysts. To further improve CoN_4_ catalysts, new design strategies and fine-tuning of CoN_4_ active sites based on fundamental understanding of the structural effects of CoN_4_ active sites on activity are needed.

MN_4_ active sites surrounded by a 14-membered-ring (14MR) structure have been proposed as a refined catalyst design because FeN_4_ active sites with a 14MR structure have been shown to exhibit significantly higher durability than those with a 16-membered-ring (16MR) structure.^8,22−24^ Considering further tuning of MN_4_ active-site structures, MN_4_ complex catalyst designs based on organic synthetic chemistry are more effective than the conventional design of MN_4_ structures embedded in a carbon matrix, which form stochastically during pyrolysis of precursors containing metal, nitrogen, and carbon, determined by thermodynamic and kinetic factors. Based on this concept, our research group developed carbon-supported FeN_4_ complex catalysts using a 14MR hexaazamacrocyclic ligand (1,14:7,8-ditethenotetrapyrido-[2,1,6-de:2′,1′6′-gh:2″,1′′,6′′-na][1,3,5,8,10,12]hexaazacyclotetradecine); these catalysts are denoted as Fe-14MR.^24,25^ Fe-14MR exhibited higher activity and durability for the ORR than the conventional Fe phthalocyanine (Pc) and Fe porphyrin catalysts, i.e., Fe complexes with 16MR ligands (hereafter denoted as Fe-16MR). Further improvements of ORR activity and durability were achieved by tuning the Fe-14MR structure by heating on a carbon support.^8^

Based on the above, CoN_4_ complexes with 14MR ligands (Co-14MR complexes) are expected to possess active-site structures distinct from those of conventional CoN_4_ catalysts, potentially enhancing their catalytic performance. Such Co-14MR complexes have not yet been explored as ORR catalysts. Very recently, one-pot gram-scale rapid synthesis of the Co-14MR complex with a 14MR hexaazamacrocyclic ligand was developed by our group.^26^ In the present study, we develop carbon-supported Co-14MR (Co-14MR/C) catalysts for the ORR and hydrogen evolution reaction (HER). Detailed structural analysis of the Co-14MR/C catalysts is conducted to unveil the structural parameters of CoN_4_ catalysts that determine their performance in the ORR and HER.

## Methods

### Catalyst Preparation

The Co-14MR complex was synthesized according to a previous report.^26^ Co-14MR/C was prepared by impregnating Co-14MR (40 mg, yellow) in water (400 mL) with Ketjen black (EC600JD, 100 mg) at 60 °C. After stirring for 2 h, the suspension was filtered. The solid was washed with water (100 mL) and then dried overnight at 110 °C to obtain Co-14MR/C-ap. CoPc/C was prepared according to the literature.^27,28^ CoPc (Sigma-Aldrich, 40 mg) was dissolved in concentrated H_2_SO_4_ (11 mL) and then Ketjen black (100 mg) was added. The suspension was stirred for 2 h at room temperature, diluted with water (Millipore water, 200 mL) in an ice bath, and then filtered. The suspension was washed with water until the pH of the filtrate reached 6–7. The residue was dried overnight at 110 °C to obtain CoPc/C. Each carbon-supported Co complex catalyst was subsequently heated at a temperature in the range of 200–900 °C under N_2_ for 2 h. The as-prepared catalysts before the heat treatment are referred to as Co-14MR/C-ap and CoPc/C-ap. The heat-treated catalysts are referred to as Co-14MR/C-T and CoPc/C-T, where T indicates the heat treatment temperature. For comparison, Fe-14MR/C-T and FePc/C-T catalysts were prepared in the same manner as the corresponding Co catalysts.^8^

### Synthesis of Deprotonated Co-14MR Complex

The reaction vessel was charged with the Co-14MR complex (0.0995 g, 0.1338 mmol) and pyridine (11 mL). Subsequently, the reaction mixture was refluxed for 7 days under air to give a dark green solution when deprotonation from NH groups of the Co-14MR complex should take place. After the reaction flask was cooled to room temperature, the precipitate was removed by filtration. The solution was concentrated by slow evaporation. This process gave dark green single crystals of deprotonated Co-14MR complex with pyridinium trifluoromethanesulfonate (0.0517 g, 0.0623 mmol, 47% yield) suitable for the single crystal X-ray diffraction study.

### Crystal Analysis for Deprotonated Co-14MR Complex

A Rigaku XtaLAB Synergy R, HyPix diffractometer system was employed to collect the crystallographic data (Cu Kα radiation). Space group *P*2_1_, *a* = 8.51550(10) Å, *b* = 22.8740(2) Å, *c* = 9.03710(10) Å, β = 92.6520(10)°, *V* = 1758.39(3) Å^3^, *Z* = 2, *T* = −100.0 °C, μ(Cu Kα) = 4.999 mm^–1^, *D*_calc_ = 1.567 g cm^3^; reflections collected/unique reflections/parameters refined: 22532/7130/514, *R*_int_ = 0.0341, final *R*_1_ = 0.0308 (*I* > 2σ(*I*)), *wR*_2_ = 0.0799 (all data), GOF = 1.038, Flack Parameter = −0.0192(18), and Hooft Parameter = −0.0313(11).

### ORR Measurements

ORR measurements were performed using an HR-500 system with an HZ-7000 bipotentiostat (Hokuto Denko). A glassy carbon rotating disk electrode (RDE) (5 mm in diameter) coated with catalyst dispersion was used as the working electrode, a reversible hydrogen electrode (RHE) was used as the reference electrode, and the Pt wire was employed as the counter electrode. A catalyst ink was prepared by dispersing catalyst (5.7 mg) in a mixture of water (200 μL), ethanol (400 μL), and 5% Nafion solution (DE521 CS type, Fujifilm Wako Pure Chemical Corporation, 100 μL). Catalyst ink (6 μL) was drop-cast on a prepolished RDE (200 μg cm_disk_^–2^). After performing two cycles of cyclic voltammetry (CV) between 1.0 and 0 V at a potential sweep rate of 50 mV s^–1^, linear sweep voltammetry (LSV) curves were recorded at 10 mV s^–1^ in a 0.5 M H_2_SO_4_ aqueous solution under N_2_-saturated conditions and then under O_2_-saturated conditions at 2500 rpm. The ORR current was obtained by subtracting the current measured under N_2_ from that collected under O_2_. After LSV measurements, potential sweeps were performed between 1.0 and 0 V at 50 mV s^–1^ for 50, 125, or 1200 cycles. After cycling, additional LSV curves were collected in the same manner as described above. All measurements were conducted at 40 °C.

The rotating ring disk electrode (RRDE) experiment was performed using a ring disk electrode with a glassy carbon disk (diameter of 5 mm) and Pt ring (5.5 mm inner diameter, 8 mm outer diameter) at 40 °C. A catalyst ink was prepared by dispersing catalyst (1.7 mg) in a mixture of water (200 μL), ethanol (400 μL), and 5% Nafion solution (100 μL). Catalyst ink (6 μL) was drop-cast on the disk electrode (60 μg cm_disk_^–2^). In the same way, a more concentrated catalyst ink was prepared using 5.7 mg of catalyst, and then 6 or 12 μL of this catalyst ink was drop-cast on disk electrodes (200 or 400 μg cm_disk_^–2^, respectively). The ring electrode was held at 1.1 V vs RHE during the ORR measurements. The collection efficiency of the ring electrodes was determined to be 0.47 using the redox couple [Fe(CN_6_)]^4–^/[Fe(CN_6_)]^3–^ according to the literature.^29^ The formation rate of H_2_O and H_2_O_2_ during the ORR are determined by (*I*_disk_ – *I*_ring_/*N*)/4 and *I*_ring_/2*N*, respectively, where *I*_disk_ and *I*_ring_ are the absolute values of the ring and disk currents at a given potential, *N* is the collection efficiency of the ring electrodes (0.47), 4 is the electron number for H_2_O formation, and 2 is that for H_2_O_2_ formation. Thus, the H_2_O_2_ yield (%H_2_O_2_) was determined by 100(*I*_ring_/2*N*)/[(*I*_disk_ – *I*_ring_/*N*)/4 + *I*_ring_/2*N*] = 200*I*_ring_/(*NI*_disk_ + *I*_ring_).^30^

### HER Measurements

The HER activity of the catalysts was measured in N_2_-saturated 0.5 M H_2_SO_4_ aqueous solutions at 40 °C after applying the same catalyst ink that was used for the ORR activity test to an RDE electrode. After ten CV cycles between 1.0 and 0 V at a potential sweep rate of 50 mV s^–1^, LSV measurements were conducted from 0.1 to −0.6 V at 10 mV s^–1^ to evaluate HER activity. Catalyst durability was assessed through chronopotentiometric measurements at a current density of −10 mA cm^–2^ to monitor potential degradation.

### Co Content Measurement

The Co content of the Co-14MR/C-T and CoPc/C-T catalysts was analyzed by electron probe microanalysis (EPMA; EPMA-1720H, Shimadzu). The electrochemically active Co site density was evaluated using a NO_2_^–^ reduction method.^31^ Co-14MR/C-T was immersed in a NaNO_2_ aqueous solution, and then NO_2_^–^ coordinated to Co active sites was reduced by sweeping the potential from 0.4 to −0.3 V vs RHE. The density of these active sites was then calculated from the cathodic current resulting from the reductive stripping of NO to NH_4_^+^ (5e^–^ transfer).

### Temperature-Programmed Desorption (TPD)

TPD experiments were carried out using a BELCAT II equipped with a BELMASS quadrupole mass spectrometer (MicrotracBEL). Co-14MR/C (50 mg) in a quartz tube was heated at 200 °C under 30 mL min^–1^ of He for 2 h. The sample temperature was increased from 200 to 800 °C at 10 °C min^–1^ under the He flow while the outlet gas was monitored with the quadrupole mass spectrometer.

### Thermogravimetry Analysis (TGA)

TGA measurement of Co-14MR/C was carried out using a TG 8121 (Rigaku corp.). The sample temperature was increased from 25 to 900 °C at 10 °C min^–1^ under a N_2_ flow.

### X-ray Photoelectron Spectroscopy (XPS)

XPS measurements of Co-14MR/C-T were carried out using a K-Alpha spectrometer (Thermo Scientific) with monochromatic Al Kα radiation, an acceleration voltage of 12 keV, beam current of 6 mA, spot size of 400 μm, and energy of 1486.6 eV. The C 1s peak at 284.8 eV was used as an internal reference to correct binding energies.

### X-ray Absorption Fine Structure Spectroscopy

Co K-edge X-ray absorption fine structure (XAFS) measurements were performed in transmission mode at the AichiSR BL5S1 beamline. The catalyst powders were shaped into pellets with a 7 mm diameter. The XAFS spectra were analyzed using Athena and Artemis software included in the Demeter package.

### Electron Microscopy

High-angle annular dark-field (HAADF) scanning transmission electron microscopy (STEM) images of Co-14MR/C-600, Co-14MR/C-700, Co-14MR/C-800, and Co-14MR/C-900 were obtained using a JEOL-ARM200F at an accelerating voltage of 60 kV. A STEM observation sample was prepared by dropping an ethanol suspension of catalyst powder onto a Cu grid with a carbon film (Quantifoil, EM Japan Co., Ltd.) and then drying.

### Evaluation of Demetalation

Catalyst ink (12 μL) prepared as described above for the RDE tests was applied to a polished glassy carbon plate to form a circle with a diameter of ∼ 0.5 cm (400 μg_cat_ cm^–2^). The catalyst-coated carbon plate was mounted in an L-shaped cell with the catalyst facing a 20 mL solution of 0.5 M H_2_SO_4_. An RHE and Pt counter electrode were placed in the cell. CV measurements were then performed for 30 cycles between 1.0 and 0 V at 50 mV s^–1^ under an N_2_ atmosphere, and then for 125 or 1200 cycles under an O_2_ atmosphere. The electrolyte solution after CV measurements was analyzed using inductively coupled plasma optical emission spectrometry (ICP-OES; Thermo iCAP 7400) to investigate Co or Fe leaching.

### Computational Details

Density functional theory (DFT) calculations for structural optimization and frequency analysis were performed with the Gaussian 16 (C.01) package^32^ at the B3LYP/def2-tzvp level with an empirical dispersion correction (D3).^33^ The solvation effect was simulated using the solvation model density (SMD) model for water.^34^ The Gibbs free energy (*G*) at 300 K for each model was calculated as *G* = *EE* + *ZPE* – *TS*, where *EE*, *ZPE*, and *TS* are the electronic energy, zero-point energy, and entropic contribution, respectively.

## Results and Discussion

### Catalyst Performance for the ORR

**Figure 1:**
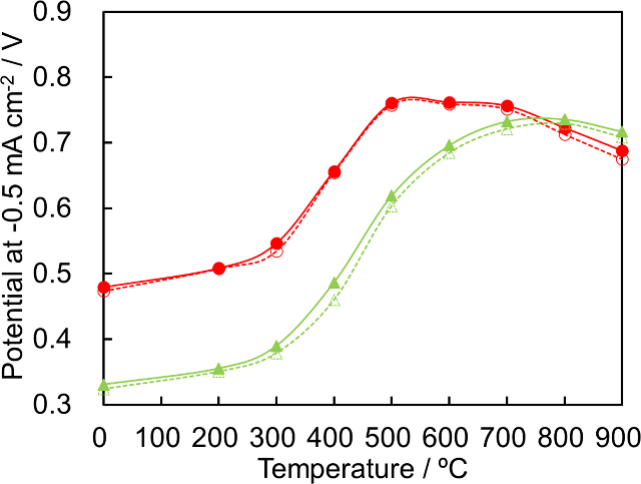
ORR onset potentials of the Co-14MR/C-T (red circles) and CoPc/C-T (green triangles) catalysts at a current density of −0.5 mA cm^–2^ as functions of the heat treatment temperature: before (solid lines and filled symbols) and after (dotted lines and open symbols) 50 potential sweeps between 1.0 and 0 V. The standard deviation of the three measurements for Co-14MR/C was within 0.3%.

The LSVs for the ORR with the Co-14MR/C-T catalysts are shown in Figure S1. Catalytic activity was evaluated based on the onset potential at a current density of −0.5 mA cm^–2^. The onset potential as a function of the heat treatment temperature is shown in Figure 1. The onset potential increased with the heat treatment temperature up to 600 °C and then decreased when the temperature exceeded 700 °C. Thus, heat treatment enhanced the ORR activity of Co-14MR/C, and Co-14MR/C-600 showed the highest activity among the Co-14MR/C catalysts. For comparison, the ORR performance of the CoPc/C-T catalysts was evaluated under the same conditions as those used to assess the Co-14MR/C-T catalysts (Figure S1). Although the catalytic performance of the CoPc/C-T catalysts improved after heat treatment, the CoPc/C-T catalysts exhibited lower activity than the Co-14MR/C-T catalysts when *T* ≤ 700 °C. The difference between the onset potentials before and after 50 cycles was evaluated as a measure of catalyst stability (Figure 1). The smaller the difference between the onset potentials, the higher the durability of the catalyst. Thus, the durability of the Co-14MR/C-T catalysts with *T* ≤ 700 °C was higher than that of the CoPc/C-T catalysts. Overall, the Co-14MR-based catalysts displayed higher activity and stability for the ORR than the conventional Co-16MR-based catalysts. This result is consistent with the comparison between Fe-14MR and Fe-16MR catalysts in our previous study.^8^

The mass activity at 0.8 V vs RHE, active site density, and turnover frequency (TOF, determined in terms of electrons per site per second) of Co-14MR/C-600 and Co-14MR/C-200 are summarized in Table 1. The active site density of Co-14MR/C-600 was similar to that of Co-14MR/C-200, but the TOF of Co-14MR/C-600 was much higher than that of Co-14MR/C-200. The mass activity can be decomposed into the active site density and TOF (mass activity = active site density × TOF), so the higher mass activity of Co-14MR/C-600 compared with that of Co-14MR/C-200 is attributed to the enhanced catalytic activity of Co active sites in the former compared with that in the latter. The Co-14MR/C catalysts exhibited higher TOFs than the CoPc/C catalysts (Table 1), indicating that the 14MR ligand raised the ORR activity of Co centers more effectively than the 16MR (Pc) ligand. When compared to Fe-14MR/C-600 and reported Fe–N–C catalysts (Table S1), the mass activity of Co-14MR/C-600 is lower due to its lower TOF and/or active site density compared to the Fe catalysts.^35^ The reason for the differences in TOF between the Co-14MR/C and Fe-14MR/C catalysts will be discussed later (see Effect of Catalyst Structure on ORR Activity). However, it is noteworthy that the mass activity of Co-14MR/C-600 was higher than that of the conventional FePc/C-600 and as high as that of Fe-14MR/C-200. Thus, our results suggest that the Co-14MR-based catalyst design can improve the ORR activity of CoN_4_ catalysts to the level of FeN_4_ catalysts.

**Table 1:** Metal Loading, Mass Activity, Active Site Density, and TOF of Co-14MR/C-200, Co-14MR/C-600, CoPc/C-200, CoPc/C-600, Fe-14MR/C-200, Fe-14MR/C-600, and FePc/C-600 Catalysts for the ORR

Catalyst	Metal loading^a^ (wt %)	Mass activity^b^ (A g^–1^)	Active site density^c^ (×10^19^ site g^–1^)	TOF^d^ (electrons site^–1^ s^–1^)	Ref
Co-14MR/C-200	2.33(10)	0.06(3)	1.99(9)	0.02	This work
Co-14MR/C-600	2.35(11)	0.81(5)	2.65(23)	0.19	This work
CoPc/C-200	2.82(9)	<0.001	1.81(7)	<0.001	This work
CoPc/C-600	2.74(8)	<0.001	2.65(27)	<0.001	This work
Fe-14MR/C-200	1.50	0.82	3.73	0.14	(8)
Fe-14MR/C-600	1.60	5.64	4.88	0.72	(8)
FePc/C-600	0.11	0.29	0.72	0.25	(8)

a Determined by EPMA.

b Evaluated from the current density at 0.8 V vs RHE.

c Determined by a NO_2_^–^ reduction method.

d Calculated by dividing the mass activity by the active site density and the Faraday constant. The errors in brackets were based on the standard deviations of [a] five and [b, c] three measurements.

The number of electrons involved in the ORR over selected Co-14MR-T and CoPc/C-T catalysts was evaluated using the RRDE method. The number of electrons involved in the ORR over the Co-14MR/C-200, CoPc/C-200, Co-14MR/C-600, and CoPc/C-600 catalysts was 2.8, 2.7, 3.4, and 3.0, respectively (Figure S2) with associated H_2_O_2_ selectivity of 61%, 67%, 31%, and 50%, respectively. Thus, the Co-14MR structure and heat treatment at 600 °C increased the number of electrons that participated in the catalytic reaction. This suggests that the 2 × 2 electron reduction pathway and/or direct 4 electron pathway were facilitated on the Co-14MR/C-600 catalyst due to the improved adsorption property for oxygen species, which will be discussed later (see Effect of Catalyst Structure on ORR Activity).^24,29,36^ Notably, the reaction electron number for Co-14MR/C-600 (3.4) is comparable to those of Fe-14MR/C-600 and Fe-14MR/C-200 (3.6).^8^ In addition, when the amount of Co-14MR/C-600 catalyst on the RDE was increased from 60 to 200 and 400 μg_cat_ cm^–2^, the number of electrons involved in the ORR further increased to 3.7 (Figure S3). Thus, Co-14MR/C-600 shows potential for practical use as a non-PGM ORR catalyst.

**Figure 2:**
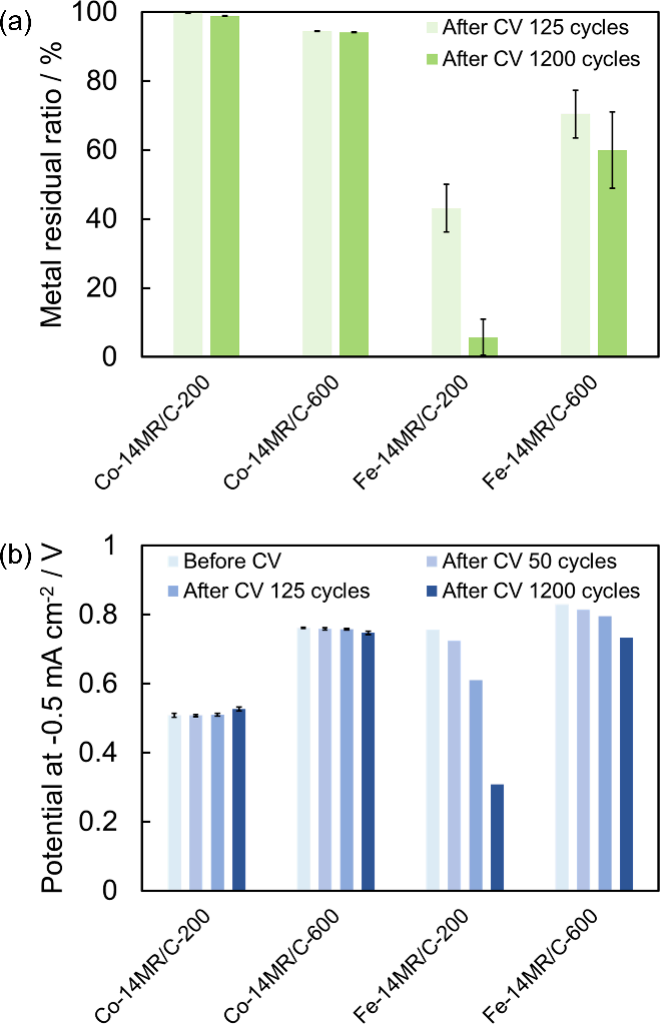
(a) Metal residual ratios of Co-14MR/C-200, Co-14MR/C-600, Fe-14MR/C-200, and Fe-14MR/C-600 after 125 and 1200 cycles. The error bars for Co and Fe catalysts were based on the standard deviations of six and two measurements, respectively. (b) The ORR onset potentials of Co-14MR/C-200, Co-14MR/C-600, Fe-14MR/C-200, and Fe-14MR/C-600 catalysts at a current density of −0.5 mA cm^–2^ before, after 50, after 125, and after 1200 potential-sweep cycles. The error bars for Co catalysts were based on the standard deviations of two measurements.

The durability of the Co-14MR/C catalysts was investigated by evaluating demetalation during the ORR. The amount of dissolved metal ions after 125 or 1200 CV cycles between 1.0 and 0 V vs RHE in O_2_-saturated aqueous H_2_SO_4_ was measured by ICP-OES. Figure 2(a) shows the metal residual ratios of Co-14MR/C-200 and Co-14MR/C-600 catalysts together with those of Fe-14MR/C-200 and Fe-14MR/C-600 catalysts after 125 and 1200 cycles (Table S2). The demetalation ratios were calculated by dividing the amount of dissolved metal by the initial amount of metal in the catalysts coated on a plate electrode, and then the metal residual ratio was determined from the demetalation ratio. The demetalation ratio of Co-14MR/C-600 was 6%, which was higher than that of Co-14MR/C-200. This indicates that Co-14MR/C-600 has slightly lower durability than that of Co-14MR/C-200. Thus, heat treatment at 600 °C enhanced the ORR activity of Co-14MR/C but lowered its durability. More importantly, compared with that of the Fe-14MR/C catalysts, the demetalation ratio of Co-14MR/C-600 was much smaller. In fact, the ORR activity of Co-14MR/C-600 surpassed that of Fe-14MR/C-600 after 1200 CV cycles (Figure 2(b) and Figures S4). Therefore, Co-14MR/C-600 shows improved ORR activity while maintaining the high durability of Co-based catalysts.

### Catalyst Performance for the HER

**Figure 3:**
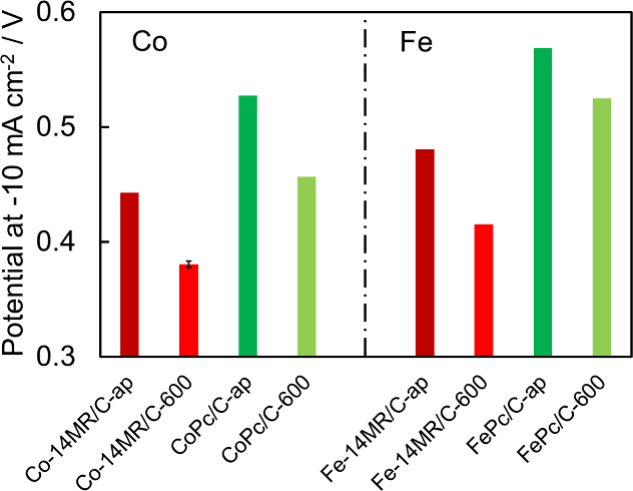
HER overpotential at a current density of −10 mA cm^–2^ over Co-14MR/C, CoPc/C, Fe-14MR/C, and FePc/C catalysts. The error bar of Co-14MR/C-600 was based on the standard deviation of two measurements.

The LSV curves for the HER on Co-14MR/C-ap, Co-14MR/C-600, Fe-14MR/C-ap, and Fe-14MR/C-600 were measured (Figure S5). For comparison, those on the CoPc/C and FePc/C catalysts were also acquired. The potential at a current density of −10 mA cm^–2^ was evaluated as the HER activity and is presented in Figure 3, where a lower potential indicates higher HER activity. The HER activity of all catalysts was improved by heat treatment at 600 °C. In addition, the Co-14MR/C and Fe-14MR/C catalysts showed higher HER activity than the corresponding 16MR catalysts, consistent with the behavior of the catalysts in the ORR. However, the order of HER activity of Co and Fe catalysts differed from that of the ORR: the Co catalysts exhibited higher activity than the Fe catalysts in the HER. Consequently, Co-14MR/C-600 was the most active catalyst among those shown in Figure 3.

The active site density and TOF at −0.4 V vs RHE of the Co and Fe catalysts are presented in Table 2. Co-14MR/C-600 exhibited the highest TOF among the catalysts, while its active site density was lower than those of the Fe-14MR/C-200 and Fe-14MR/C-600 catalysts and similar to that of the CoPc/C-600 catalyst. Therefore, the highest HER performance of Co-14MR/C-600 is accounted for by its high TOF; namely, it has the highest catalytic activity per active center of the investigated catalysts. TOFs of the Fe-14MR/C catalysts were lower than those of the FePc/C catalysts. Thus, the 14MR ligand was effective for the Co center but not for the Fe center with respect to HER activity.

**Table 2:** Mass Activity, Active Site Density, and TOF of Co and Fe Catalysts for the HER

Catalyst	Mass activity^a^ (A g^–1^)	Active site density^b^ (×10^19^ site g^–1^)	TOF^c^ (electrons site^–1^ s^–1^)
Co-14MR/C-200	15.1	1.99(9)	4.7
Co-14MR/C-600	79.9(65)	2.65(23)	18.9
CoPc/C-200	2.8	1.81(7)	1.0
CoPc/C-600	14.2	2.65(27)	3.3
Fe-14MR/C-200	8.4	3.73	1.4
Fe-14MR/C-600	35.0	4.88	4.5
FePc/C-200	3.9	0.48	5.1
FePc/C-600	6.3	0.72	5.4

a Evaluated from the current density at −0.4 V vs RHE.

b Determined by a NO_2_^–^ reduction method.

c Calculated by dividing the mass activity by the active site density and the Faraday constant. The errors in brackets were based on the standard deviation of [a] two and [a] three measurements.

**Figure 4:**
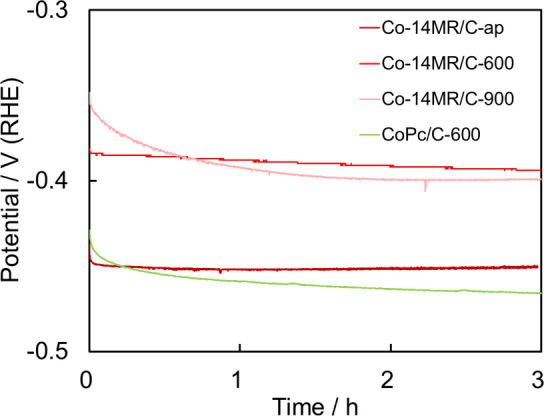
Time-dependent overpotential curves of Co-14MR/C-ap, Co-14MR/C-600, Co-14MR/C-900, and CoPc/C-600 at −10 mA cm^–2^.

The durability of the Co-14MR/C-ap, Co-14MR/C-600, Co-14MR/C-900, and CoPc/C-600 catalysts for the HER was evaluated by monitoring the potential at −10 mA cm^–2^ for 3 h, as shown in Figure 4. The Co-14MR/C-600 and Co-14MR/C-ap catalysts exhibited slight decreases in potential after reaction for 3 h, whereas CoPc/C-600 showed a considerable decrease in potential. This indicates that the Co-14MR/C catalysts have higher durability than the conventional Co-16MR/C catalysts in the HER. Co-14MR/C-900 exhibited higher HER activity than Co-14MR/C-600 in their initial LSV curves (Figure S6). However, the stability of Co-14MR/C-900 was low: as shown in Figure 4, the potential at −10 mA cm^–2^ for Co-14MR/C-900 decreased markedly and was lower than that of Co-14MR/C-600 after just 0.6 h.

### Catalyst Structure

**Figure 5:**
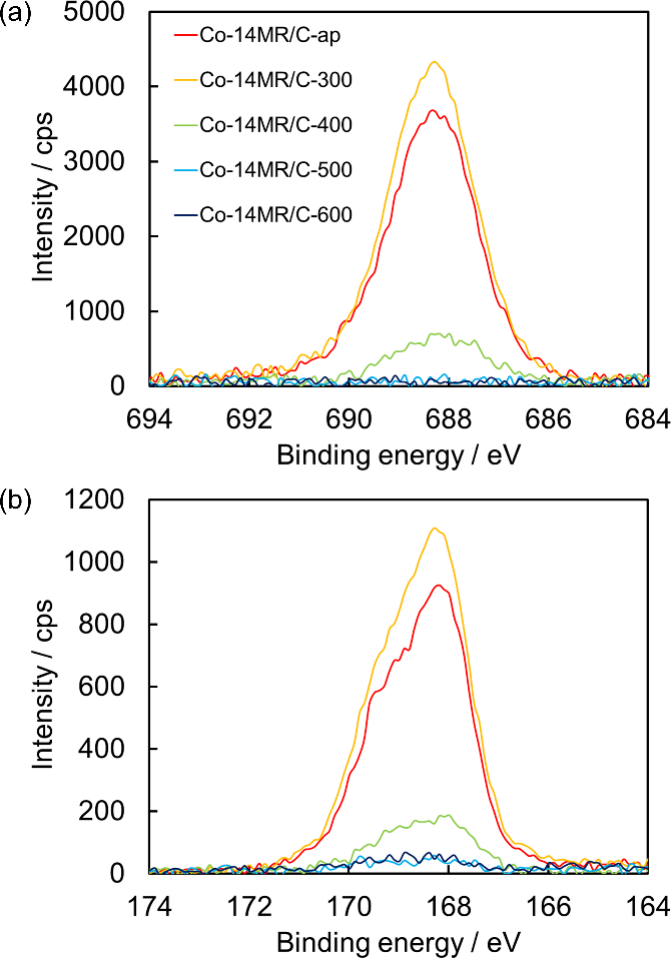
(a) F 1s and (b) S 2p XPS of Co-14MR/C-T specimens.

The Co-14MR complex used to prepare the Co-14MR/C catalyst has two CF_3_SO_3_^–^ counteranions per Co-14MR molecule, so the structural change of Co-14MR/C induced by heat treatment was investigated in terms of CF_3_SO_3_^–^ counteranions by analyzing F 1s and S 2p XPS profiles of the Co-14MR/C-T catalysts, as shown in Figure 5(a) and (b), respectively. The intensity of F 1s and S 2p XPS peaks decreased greatly after heat treatment at 400 °C and almost disappeared following heat treatment at 500 °C. Therefore, the CF_3_SO_3_^–^ counteranion desorbs from 400 °C and the Co-14MR/C-T catalysts treated at ≥ 500 °C have almost no CF_3_SO_3_^–^ counteranions. The desorption of counteranions was also supported by the results of a TPD experiment (Figure S7(a)). A TGA measurement of Co-14MR/C (Figure S7(b)) showed a weight loss of 10.1% between 360 and 600 °C, which was in good agreement with the expected 11.6% weight loss due to the desorption of triflate anions from Co-14MR/C-ap. Thus, the TGA confirmed the desorption of counteranions during the 600 °C treatment. Above 700 °C, further weight loss was observed, which is attributed to the decomposition of the Co complex structure, leading to the formation of Co metal nanoparticles.

**Figure 6:**
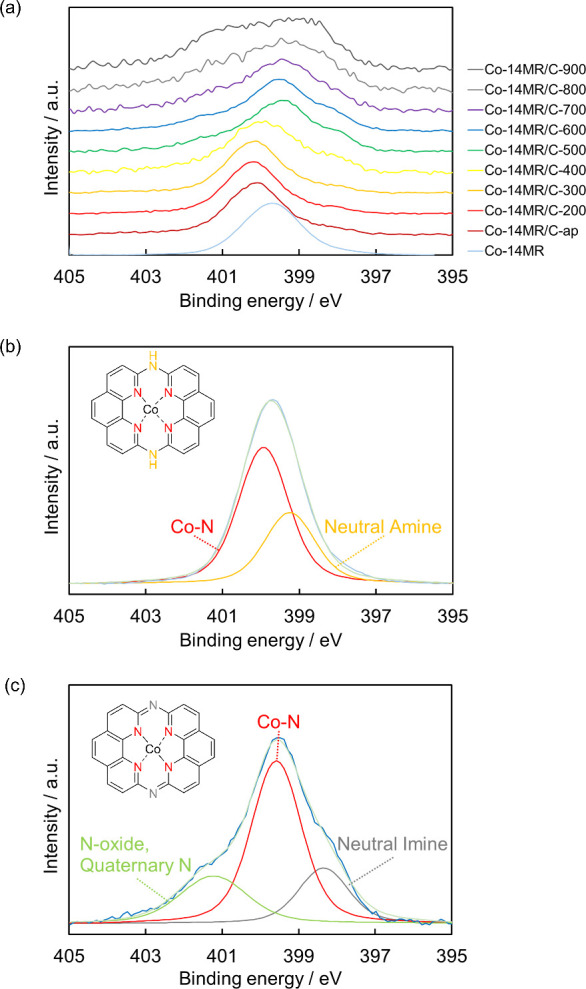
N 1s XPS of (a) Co-14MR/C-T, (b) Co-14MR complex, and (c) Co-14MR/C-600 specimens.

Figure 6(a) presents the N 1s XPS of the Co-14MR complex and Co-14MR/C-T catalysts. As shown in Figure 6(b), the N 1s XPS of Co-14MR complex was deconvoluted into the two peaks at 399.6 and 400.3 eV that were assignable to neutral amine and N coordinated to Co (Co–N), respectively. The ratios of the neutral amine and Co–N species were 34% and 66%, respectively, which aligns with the ligand structure of the Co-14MR complex.^37,38^ Co-14MR/C-ap and Co-14MR/C-200 showed similar spectral shapes but a slight peak energy shift in comparison with that of the Co-14MR complex. This energy shift would be caused by the interaction of the Co-14MR complex with the carbon support. Heat treatment caused a change in the N 1s spectra of the samples. Co-14MR/C-600 displayed peaks at 398.4, 399.6, and 401.2 eV, which are attributable to neutral imine, Co–N, N-oxide/quaternary N, respectively, as presented in Figure 6(c).^37,39^ This spectral change suggested that the amine groups in the Co-14MR complex are converted to imine groups by deprotonation of the 14MR ligand during heat treatment at 600 °C (Figure 6(c)). In addition, the N-oxide and quaternary N species are likely formed by partial decomposition of the Co-14MR complex during heat treatment. The shoulder peak ascribed to the N-oxide and quaternary N species grew considerably at ≥ 700 °C, indicating substantial decomposition of the Co-14MR structure above 700 °C.

The composition ratios of Co, N, and C, as well as the N/Co ratio of the Co-14MR/C-T catalysts were evaluated from the relative areas of N 1s and Co 2p XPS peaks (Table S3). The N/Co ratio of the Co-14MR/C-T was approximately 6 up to 600 °C and then decreased at ≥ 700 °C, suggesting that the Co-14MR complex structure is maintained at 600 °C but decomposes above 700 °C. In addition, the Co and N composition ratios decreased above 700 °C (Table S3), accompanied by a decrease in sample weight as shown by the TGA (Figure S7(b)). These results suggest that the 14MR ligand structure is maintained up to 600 °C but decomposes with material loss due to desorption of decomposed materials above 700 °C. Based on these results, the structure presented in Figure 6(c), later denoted as the Co-14MR-B structure, is proposed to exist in Co-14MR/C-600. The proposed structure contains imine groups formed by the deprotonation of amines in the precursor Co-14MR complex, accompanied by the desorption of counteranions during the 600 °C treatment, resulting in the formation of a coordination complex between Co^2+^ and 14MR^2–^.

**Figure 7:**
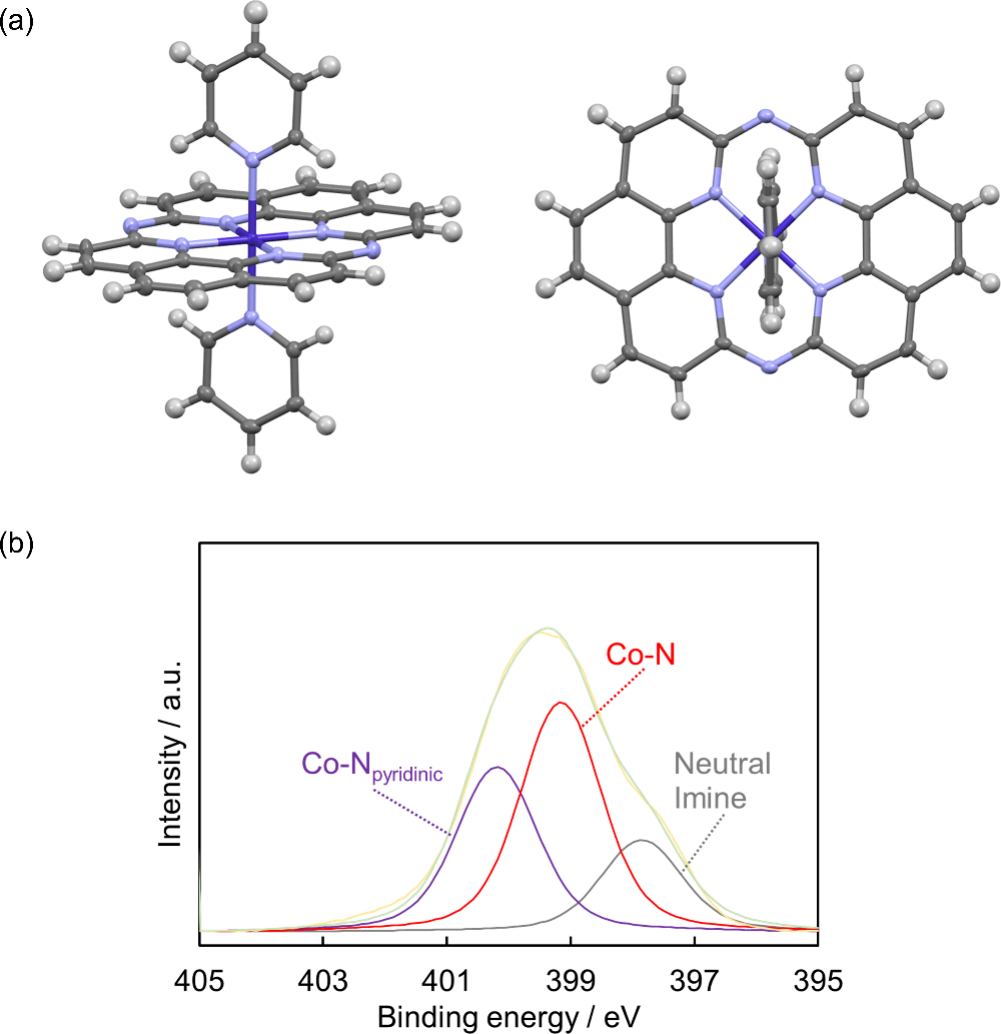
(a) Side view (left) and top view (right) of the crystal structure of deprotonated Co-14MR complex with thermal ellipsoids at the 50% probability level. (Co: navy, C: gray, N: pale blue, H: pale gray. The counteranion CF_3_SO_3_^–^ and the crystallization solvent pyridine are omitted for clarity.) (b) N 1s XPS of deprotonated Co-14MR complex.

To obtain further experimental evidence for the formation of the proposed structure in Figure 6(c), it was prepared by deprotonating the Co-14MR complex with pyridine. Figure 7(a) shows the single-crystal X-ray structure of the deprotonated Co-14MR complex after the pyridine treatment, which exhibits the same structure as the proposed one in Figure 6(c), with pyridine as axial ligands. Thus, pyridine not only served as a base for deprotonating the amine groups in the precursor Co-14MR complex but also as an axial ligand to form the structure. Figure 7(b) shows the N 1s XPS profile of the deprotonated Co-14MR complex, which can be deconvoluted into three peaks corresponding to the imine nitrogen in 14MR (397.9 eV), nitrogen in 14MR coordinated to Co (399.2 eV), and nitrogen in pyridine as axial ligands (400.2 eV). The results confirm the assignment of N 1s XPS peaks in Co-14MR/C-600 and thus the formation of the deprotonated Co-14MR structure in Co-14MR/C-600.

The stability of deprotonated Co-14MR structure in Co-14MR/C-600 during the ORR was evaluated by comparing the N 1s XPS profiles of Co-14MR/C-600 before and after 50 ORR cycles (Figure S8 and Table S4). A portion of the imine groups (39%) in Co-14MR/C-600 was protonated; however, the protonation of imines is considered to lead to protonated imine species rather than amines. More importantly, a large portion of the imine groups (61%) was retained after the ORR, indicating that the deprotonated structure is considered as the main active site structure in Co-14MR/C-600. The stability of the deprotonated Co-14MR structure against protonation is possibly attributable to the coordination complex between Co^2+^ and the 14MR^2–^ ligand, which is formed via desorption of the counterions and the amine protons from the precursor Co-14MR complex during the heat treatment.

**Figure 8:**
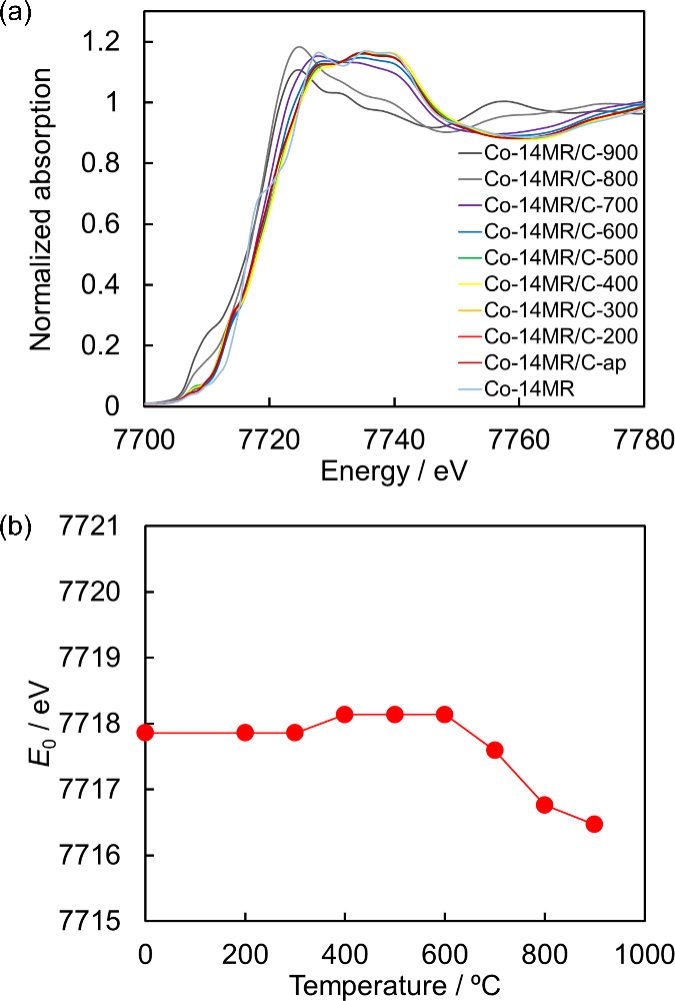
(a) Co K-edge XANES spectra of Co-14MR/C-T specimens and the Co-14MR complex. (b) *E*_0_ values of Co-14MR/C-T specimens as a function of heat treatment temperature.

The changes in the oxidation state and local structure of Co in Co-14MR/C induced by heat treatment were analyzed using Co K-edge XAFS spectroscopy. Figure 8(a) and Figure S9 display the Co K-edge X-ray absorption near-edge structure (XANES) spectra of the Co-14MR/C-T catalysts. In Figure 8(b), the absorption edge energy (*E*_0_), which is defined as the energy at a normalized absorbance of 0.5, is plotted against heat treatment temperature for the Co-14MR/C-T catalysts. *E*_0_ of the Co-14MR/C-T catalysts with T ≤ 600 °C suggested that the oxidation state of Co in the samples is 2+ (Figure S10). *E*_0_ decreased at ≥ 700 °C. In addition, the spectra of the Co-14MR/C-800 and Co-14MR/C-900 catalysts showed a shoulder peak at 7710 eV consistent with the spectrum of Co foil (Figure S9). Therefore, the Co-14MR structure decomposes to form Co metal particles at ≥ 700 °C. The XANES results are consistent with those of XPS.

Figure 8(b) shows a slight increase in *E*_0_ as the heat treatment temperature rises from 300 to 600 °C. Meanwhile, Figure 6 shows that the N 1s XPS peak corresponding to Co–N shifts to a lower binding energy with heat treatment. These results suggest that electron transfer from Co to N occurs in the complex structure of Co-14MR/C-600. It is reasonable to consider that the resulting electronic state of the CoN_4_ active center alters the adsorption energy of oxygen species, thereby leading to improved ORR activity in Co-14MR/C-600.

**Figure 9:**
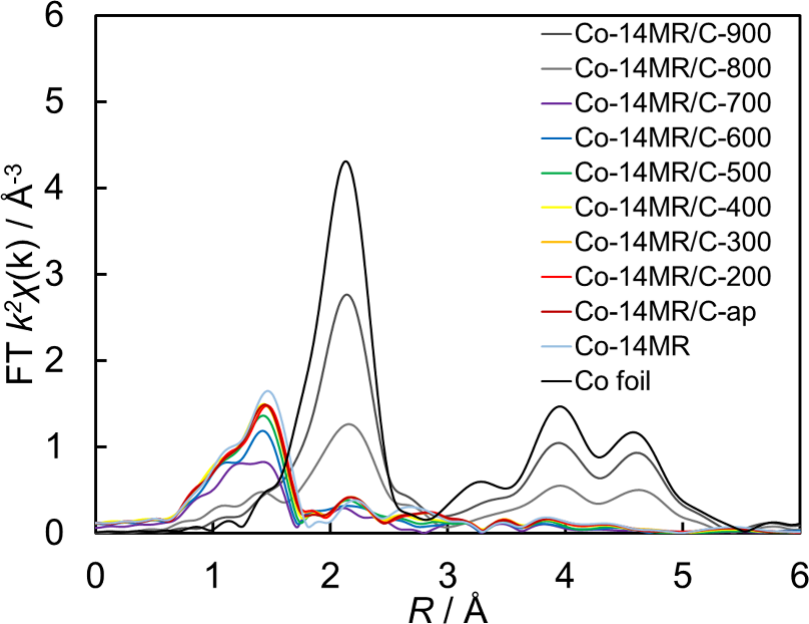
Co K-edge FT EXAFS spectra of Co-14MR/C-T catalysts, Co-14MR complex, and Co foil.

Changes in the local structure of the Co-14MR/C-T catalysts induced by heat treatment were investigated by analysis of Co K-edge Fourier transform (FT) extended X-ray absorption fine structure (EXAFS) spectra, which are shown in Figure 9 and Figure S11. The peak at 1.4 Å was assigned to the scattering of Co–N and Co–O of a Co complex and that at 2.2 Å was consistent with Co–Co of Co metal. The decrease of the first-shell peak intensity at 500–600 °C can be explained by a slight change of the CoN_4_ local structure caused by heat treatment. The coordination number of Co–N(O) did not change much up to 600 °C (Table S5). In other words, the CoN_4_ local structure was maintained at ≤ 600 °C. At ≥ 700 °C, the decrease of the peak intensity at 1.4 Å and increase of that at 2.2 Å suggested the decomposition of the CoN_4_ local structure and formation of Co metal.

**Figure 10:**
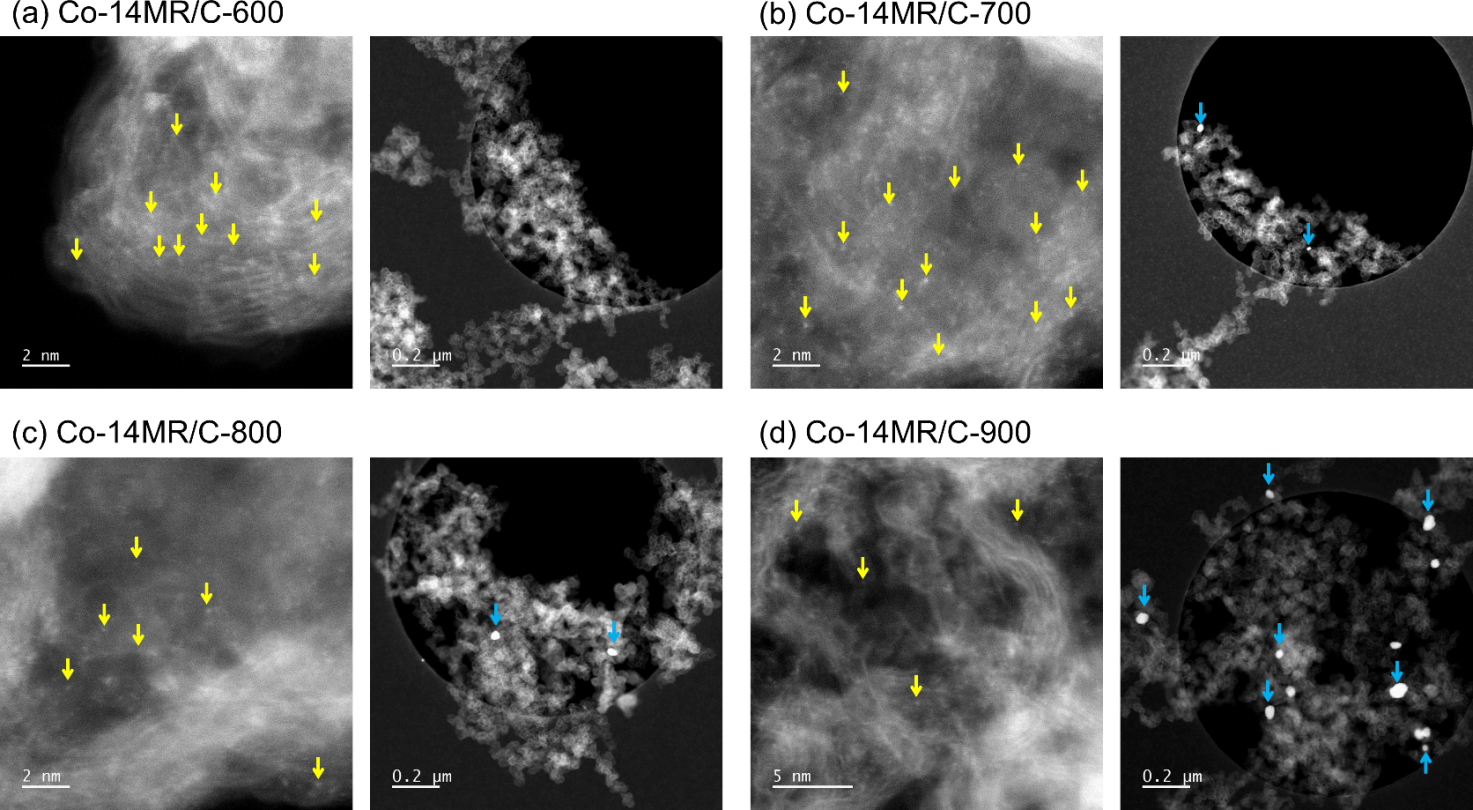
Typical HAADF-STEM images of (a) Co-14MR/C-600, (b) Co-14MR/C-700, (c) Co-14MR/C-800, and (d) Co-14MR/C-900. Some of single Co atoms and Co nanoparticles are indicated by yellow and light blue arrows, respectively.

Figure 10 shows typical HAADF-STEM images of Co-14MR/C-600, Co-14MR/C-700, Co-14MR/C-800, and Co-14MR/C-900. In Co-14MR/C-600, atomically isolated Co species were observed on carbon without the formation of Co nanoparticles, whereas Co nanoparticles were observed in Co-14MR/C-700. Furthermore, Co nanoparticles with larger particle sizes became more prominent in Co-14MR/C-800 and Co-14MR/C-900. Although single Co atoms were still observed in Co-14MR/C-800 and Co-14MR/C-900, the majority of Co species are regarded as being present in Co nanoparticles, as indicated by their FT-EXAFS spectra (average data) which showed Co–Co scattering peak rather than Co–N scattering peak (Figure 9). These results confirm the structural evolution of Co-14MR/C during heat treatment, indicating that the CoN_4_ structure is retained up to 600 °C, but gradually decomposes to form Co nanoparticles at 700 °C and higher temperatures.

The XANES and EXAFS results and the HAADF-STEM observation confirmed that the majority of the Co-14MR complex on carbon supports changed to its imine form (Figure 6(c)) upon heat treatment at 600 °C and maintained the CoN_4_ structure. The Co–N bond length was not affected much by this structural change. The Co–N bonds of Co-14MR/C-T treated at ≤ 600 °C were shorter than those of the CoPc/C-600 catalyst (Table S6). Therefore, the Co-14MR/C-T catalysts have a smaller CoN_4_ moiety than the conventional Co-16MR/C-T catalysts. To further investigate the CoN_4_ structure in the Co-14MR/C-T catalysts, the Co K-edge XANES spectra were revisited (Figure 8(a) and Figure S9). All the Co-14MR/C-T catalysts showed a small pre-edge peak, suggesting the Co species possessed an octahedral structure with relatively high symmetry. More importantly, it was found that the pre-edge peak intensity slightly increased upon heat treatment at 400–600 °C. This suggests that heat treatment caused slight distortion of the CoN_4_ structure in the Co-14MR/C-T catalysts. Overall, the compact CoN_4_ structure of Co-14MR/C-T was slightly distorted and the amine groups in the ligand were converted to imine groups by heat treatment at 400–600 °C.

### Effect of Catalyst Structure on ORR Activity

In acidic media, the ORR proceeds through the following elementary reactions:
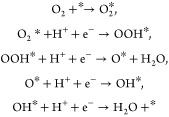
Here, * represents the catalytic active site. An O_2_ molecule is first adsorbed on the catalyst surface to form adsorbed O_2_ (O_2_*), followed by the successive addition of four electrons (e^–^) and four protons (H^+^) over four steps, with the reaction proceeding via adsorbed OOH (OOH*), O (O*), and OH (OH*) intermediates. The adsorption free energy (Δ*G*) for the formation of these intermediates serves as an indicator of the reaction barriers.^40^

**Figure 11:**
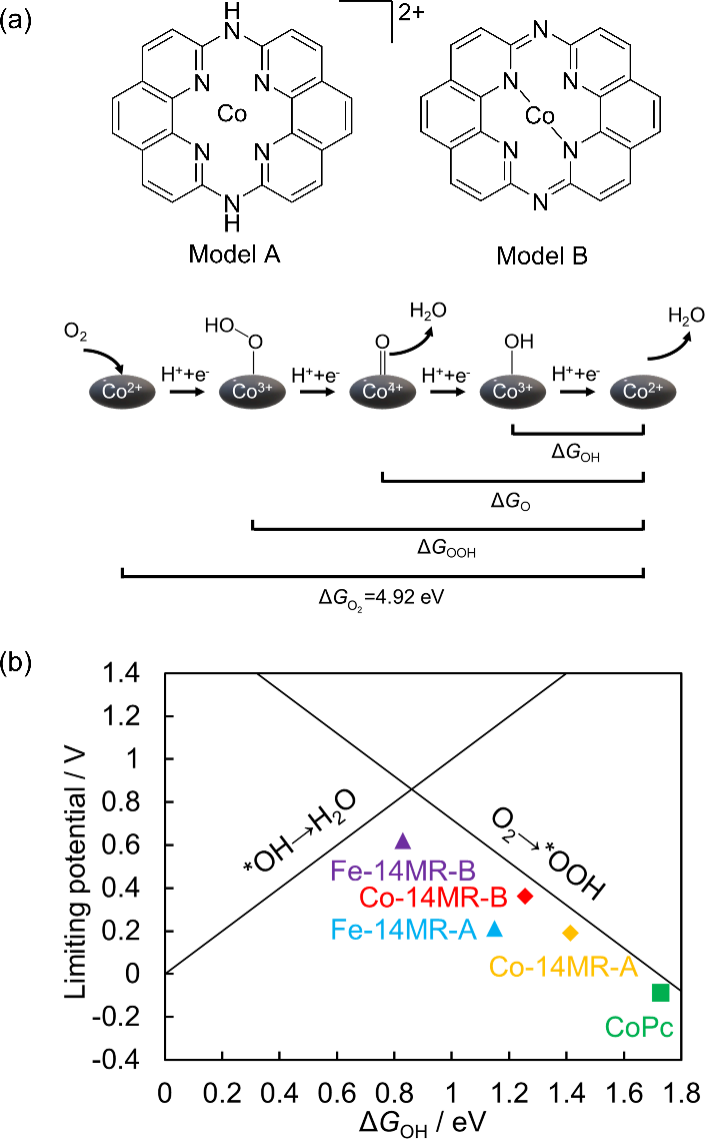
(a) Models and ORR pathway considered in the DFT simulation. (b) Relationship between the limiting potential and Δ*G*_OH_ values for Co-14MR-A, Co-14MR-B, CoPc, Fe-14MR-A, and Fe-14MR-B overlaying a volcano-type plot drawn based on previous theoretical calculations.^42^

The Co-14MR structures formed before and after heat treatment are presented in Figure 11(a) as Co-14MR-A and Co-14MR-B, respectively. The above experimental results demonstrated that Co-14MR-B has superior ORR activity to Co-14MR-A. In addition, the Co-14MR/C-T catalysts displayed higher ORR activity than the CoPc/C-T catalysts. To investigate the origin of this structural effect, the adsorption energies of various oxygen species during the ORR reaction were evaluated using a DFT calculation based on the computational hydrogen electrode model.^41^ In this calculation, Co^2+^ without adsorbed oxygen species was assigned as the initial species of the ORR and Co^3+^-OOH, Co^4+^=O, and Co^3+^–OH were designated as intermediate species (Figure 11(a)). It should be noted that adsorption energies of possible oxygen species formed during the ORR via 4 electron reduction path were evaluated in order to investigate the reason for the order of the ORR activity, although the ORR over Co-14MR/C is considered to contain 2 × 2 electron reduction mechanism. Structural optimization calculations for the four Co species with all possible spin states were performed to determine the most stable structure for each species. Then, the adsorption free-energy changes of oxygen species, i.e., Δ*G*_OOH_, Δ*G*_O_, and Δ*G*_OH_, were evaluated based on the most stable structures. The energy difference before and after the four-electron ORR was determined to be 4.92 eV (4 × 1.23 eV), which is defined as Δ*G*_O2_. Adsorption energies are presented in Table S7. Using these adsorption free-energy change data, the free-energy change associated with each reaction step was evaluated to identify the rate-determining step and limiting potentials of the ORR. The limiting potentials are listed in Table S7. The limiting potential increased in the order of Co-14MR-B > Co-14MR-A > CoPc, which agreed with the ORR activity observed experimentally.

Figure 11(b) shows the limiting potentials for Co-14MR-A, Co-14MR-B, and CoPc as a function of Δ*G*_OH_, overlapping the volcano-shaped plot based on previous theoretical calculations using the computational hydrogen electrode model.^42^ The calculation results for Co-14MR-A, Co-14MR-B, and CoPc followed the slope of the volcano plot, more specifically, the right slope of the plot, which reveals that O_2_ activation is the rate-determining step. Therefore, it is concluded that the 14MR ligand structure and structural change of Co-14MR induced by heat treatment provided a catalyst that facilitated the O_2_ activation step and thus increased the ORR activity. Moreover, the approach of Co-14MR-B to the top of the volcano plot indicates an increase in the OOH adsorption energy, which leads to the increase in the electron number described above (see Catalyst Performance for the ORR).

In our previous study, the model structures of Fe-14MR, namely Fe-14MR-A and Fe-14MR-B, which correspond to Co-14MR-A and Co-14MR-B, respectively, were investigated, and their data are also shown in Figure 11(b). All the data for the Co and Fe complex structures are aligned along the right slope of the volcano plot. Thus, the order of catalytic activity, more specifically the TOF, can be explained by the Δ*G*_OH_. Furthermore, the higher TOF of Fe-14MR/C-600 compared to that of Co-14MR/C-600 (Table 1) is attributable to the smaller Δ*G*_OH_ of Fe-14MR/C-600, in other words, its higher activity for the O_2_ activation step. The higher TOF combined with the greater active site density of Fe-14MR/C-600 accounts for its higher mass activity compared to Co-14MR/C-600 (Table 1).

### Effect of Catalyst Structure on HER Activity

**Figure 12:**
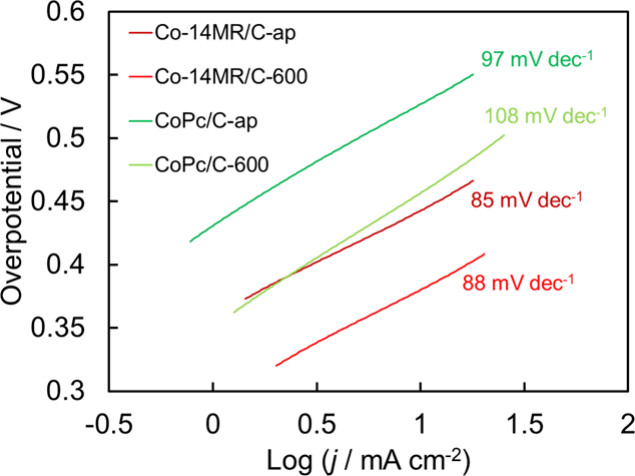
Tafel plots for the HER over Co-14MR/C-ap, Co-14MR/C-600, CoPc/C-ap, and CoPc/C-600 catalysts.

Prior to investigating the effect of catalyst structure on the HER, the rate-determining step was identified as follows. The HER is a two-electron transfer process (2H^+^ + 2e^–^ → H_2_) that occurs at the cathode during water electrolysis. Under acidic conditions, the HER proceeds through the following elementary reactions:
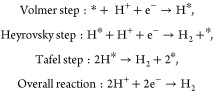
Here, H* indicates a hydrogen intermediate adsorbed on the catalyst. The HER process typically follows either the Tafel–Volmer or Heyrovsky–Volmer reaction pathways.^43−45^ The HER mechanism can be identified by the Tafel slope derived from the HER curve.^46^ If the Volmer step is the rate-determining step, the Tafel slope is approximately 120 mV dec^–1^ regardless of whether H_2_ is produced by the Heyrovsky or Tafel step. If the Heyrovsky step is the rate-determining step in the Heyrovsky-Volmer reaction, the Tafel slope becomes approximately 40 mV dec^–1^. If the Tafel step is the rate-determining step in the Tafel–Volmer reaction, the Tafel slope should be approximately 30 mV dec^–1^. Figure 12 shows the Tafel plots for the Co-14MR/C-ap and Co-14MR/C-600 catalysts, together with those of the CoPc/C-ap and CoPc/C-600 catalysts. All the Tafel slope values were within 85–108 mV dec^–1^, which are intermediate between the reference values for the Tafel slopes, suggesting contribution of multiple steps to the overall HER kinetics. The Co complex catalysts have isolated Co active sites, so it would be difficult to promote the Tafel reaction, which needs two adjacent H* to form H_2_. Thus, the HER using these Co catalysts likely involves the Heyrovsky–Volmer reaction mechanism, with contributions from both steps to the overall HER kinetics. A smaller Tafel slope suggests a greater contribution from the Heyrovsky step. Accordingly, the Heyrovsky step contributes more to the HER over the Co-14MR/C catalysts (Tafel slope: 85–88 mV dec^–1^) than over the CoPc/C catalysts (Tafel slope: 97–108 mV dec^–1^). A larger contribution of the Heyrovsky step implies stronger hydrogen adsorption. Thus, the higher catalytic activity of the Co-14MR/C catalysts compared to the CoPc/C catalysts can be attributed to the increased hydrogen adsorption energy.

The hydrogen adsorption energy (Δ*G*_H_) for Co-14MR-A, Co-14MR-B, and CoPc with Co^2+^ in a low-spin state was evaluated by DFT calculations; the results are presented in Table 3. The Δ*G*_H_ values for these models were greater than zero, indicating that hydrogen adsorption is weak. The Δ*G*_H_ value decreased in the order Co-14MR-B < Co-14MR-A < CoPc, which is consistent with the order of HER activity of Co-14MR/C-600 > Co-14MR/C-ap > CoPc/C-ap. In addition, the stronger hydrogen adsorption of the Co-14MR catalysts compared to CoPc is consistent with the above insights gained from the comparison of Tafel slopes. Since hydrogen adsorption energy is related to the electronic structure of the active center, these results indicate that the change in the electronic structure of the Co active center, induced by the ligand structure and heat treatment, enhances hydrogen adsorption energy and thus improves the TOF for the HER.

**Table 3:** Calculated Adsorption Free Energies of Hydrogen of Co-14MR-A, Co-14MR-B, and CoPc

Model	Free energy of hydrogen Δ*G*_H_ at 300 K/eV (Co spin state)
A	0.706 (1/2)
B	0.700 (1/2)
CoPc	1.043 (1/2)

### Effect of Catalyst Structure on Durability

Our previous studies on Fe-14MR/C-T catalysts suggested that the Fe–N bond length and symmetry around the Fe center are the major factors controlling catalyst durability during the ORR in acidic media.^8,24^ The short (i.e., strong) Fe–N bond provided by the compact 14MR ligand moiety leads to high durability. Additionally, the high symmetry (limited distortion) around the Fe center in Fe-14MR, with the Fe ion in the plane of the 14MR ligand, also enhance durability. This is because metal ions are more difficult to dissociate from ligands in complexes with strong Fe–N bonds and symmetrical structures than from those with weak Fe–N bonds and distorted structures. Based on these findings, the effects of Co–N bond length and symmetry around the Co center on catalyst durability were investigated. The Co–N bonds of Co-14MR/C-200 and Co-14MR/C-600 were shorter than those of CoPc/C-600 (Tables S5 and S6), which accounts for the higher durability of Co-14MR/C-200 and Co-14MR/C-600 catalysts than that of CoPc/C-600. Regarding the effect of symmetry on catalyst durability, Co-14MR/C-200 exhibited higher symmetry than Co-14MR/C-600 (Figure 8(a)), which is responsible for the superior durability of the former over the latter (Figure 1 and 2(a) and Table S2). Therefore, the order of durability of the Co complex catalysts can be explained by the Co–N bond length and the symmetry around Co: the shorter the Co–N bond and the higher the symmetry around the metal center, the greater the catalyst durability. It should also be noted that Co-14MR/C-600 demonstrated enhanced activity but decreased durability. This trade-off between activity and durability is frequently observed in catalysts; however, as reported previously, Fe-14MR/C-600 exhibited both enhanced activity and durability compared to Fe-14MR/C-200, suggesting that appropriate catalyst design can overcome this trade-off.^8^

**Figure 13:**
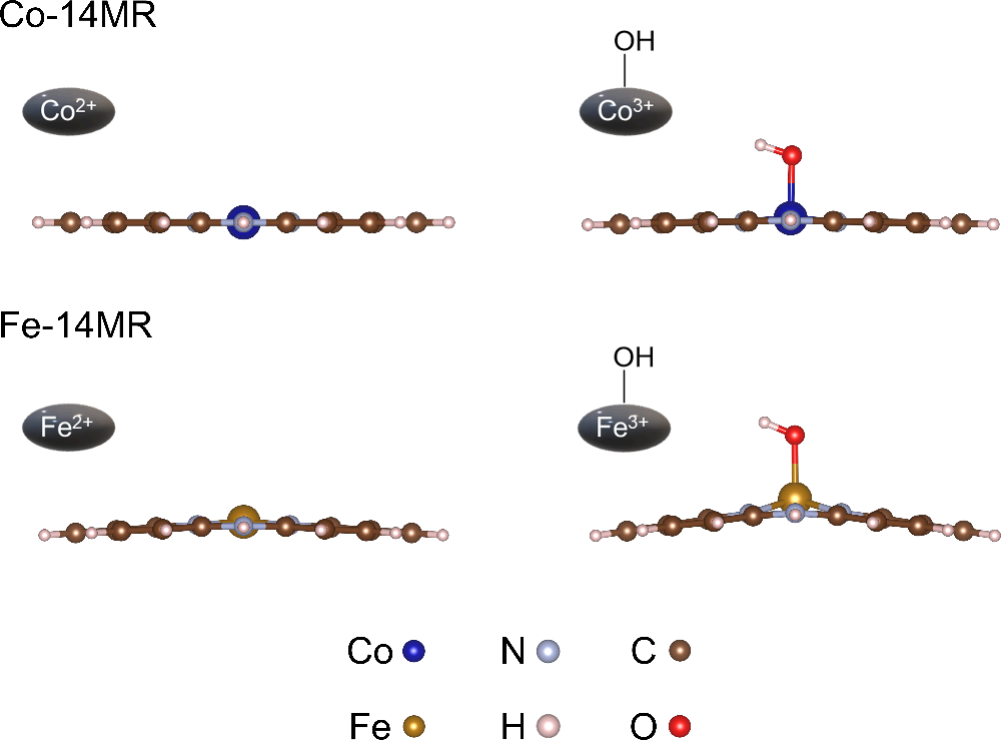
Side views of the optimized structures of Co-14MR (top) and Fe-14MR (bottom) complexes without (left) and with (right) OH^–^ as an axial ligand.

Figure 13 shows side views of the Co-14MR and Fe-14MR complexes after optimization using DFT calculations. The Co-14MR complex has a flatter structure than the Fe-14MR complex. In other words, the Co-14MR complex has higher symmetry around the metal center than Fe-14MR. In addition, the FT-EXAFS results (Table S6) showed that the Co-14MR complex has shorter metal-N bonds than the Fe-14MR complex. Accordingly, both the higher symmetry/planarity of the active metal center in Co-14MR/C and the short metal-N bond are responsible for its superior durability to that of the Fe-14MR/C catalysts. Overall, it is concluded that the short Co–N bond length and high symmetry of Co-14MR/C catalysts are important factors determining their high stability in electrochemical reactions under harsh acidic conditions.

## Conclusions

This study demonstrated that Co-14MR/C catalysts outperform conventional Co-16MR/C catalysts in both the ORR and HER under acidic conditions. Heat treatment of Co-14MR/C at 600 °C markedly enhanced its ORR and HER activities. The enhanced catalytic activity of the Co-14MR/C catalysts resulted from the optimization of adsorption energies of oxygen and hydrogen intermediates caused by structural changes of the Co active site induced by the 14MR ligand as well as the modification of the Co-14MR structure by heat treatment. The Co-14MR/C catalysts exhibit higher resistance to demetalation in acidic conditions than the CoPc/C and Fe-14MR/C catalysts. The high durability of the Co-14MR/C catalysts is ascribed to their short Co–N bonds and high symmetry around the Co center. These results suggest that the Co-14MR structure is a potential basis for the development of highly durable and efficient non-PGM catalysts for fuel cells and water-splitting.

## Supporting Information Available

LSV curves, demetalation ratios, TPD and TGA profiles, XPS spectra, XPS analysis results, XAFS spectra, EXAFS curve fitting results, X-ray absorption energies, and DFT calculation results (PDF)
