# Supplementary material for: Fourteen-Membered Macrocyclic Cobalt Complex Structure as a Potential Basis for Durable and Active Non-platinum Group Metal Catalysts for Oxygen Reduction and Hydrogen Evolution Reactions
Source: J Am Chem Soc. 2025 Apr 25;147(18):15377–88. doi: 10.1021/jacs.5c01306 (PMC12063610; doi:10.1021/jacs.5c01306)
Supplement: Supplementary file 1 — ja5c01306_si_001.pdf [file ja5c01306_si_001.pdf]

Supporting Information

Fourteen-Membered Macrocyclic Cobalt Complex Structure as a Potential Basis for Durable and Active Non-Platinum Group Metal Catalysts for Oxygen Reduction and Hydrogen Evolution Reactions

*Zhiqing Feng,[a] Junya Ohyama,[*b,c] Soutaro Honda,[a] Yasushi Iwata,[b] Keisuke Awaya,[b] Masato Machida,[b,c] Masayuki Tsushida,[d] Ryota Goto,[e] Takeo Ichihara,[e] Makoto Moriya,[f,g] Yuta Nabae[*h]*

[a] Graduate School of Science and Technology, Kumamoto University, 2-39-1 Kurokami, Chuo-ku, Kumamoto, 860-8555, Japan.

[b] Faculty of Advanced Science and Technology, Kumamoto University, 2-39-1 Kurokami, Chuo-ku, Kumamoto 860-8555, Japan.

[c] Institute of Industrial Nanomaterials (IINa), Kumamoto University, 2-39-1 Kurokami, Chuo-ku, Kumamoto 860-8555, Japan.

[d] Technical Division, Kumamoto University, 2-39-1 Kurokami, Chuo-ku, Kumamoto 860-8555, Japan.

[e] Corporate R&D, Asahi Kasei Corporation, 2767-11 Niihama, Shionasu, Kojima, Kurashiki, Okayama 711-8510, Japan.

[f] College of Science, Academic Institute, Shizuoka University, 836 Ohya, Suruga-ku, Shizuoka 422-8529, Japan.

[g] Research Institute of Green Science and Technology, Shizuoka University, 836 Ohya, Suruga-ku, Shizuoka 422-8529, Japan.

[h] Department of Materials Science and Engineering, Institute of Science Tokyo, 2-12-1 S8-26, Ookayama, Meguro-ku, Tokyo 152-8552, Japan.

ohyama@kumamoto-u.ac.jp (JO), nabae.y.aa@m.titech.ac.jp (YN)

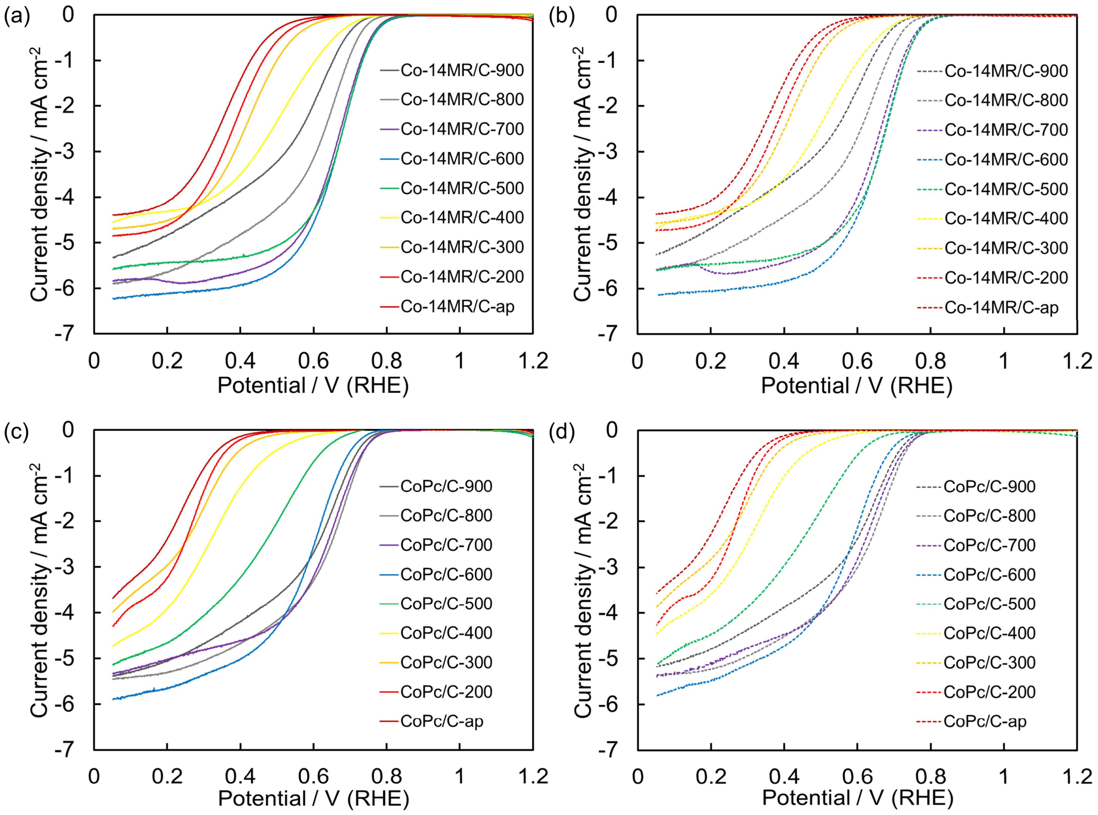

Figure S1. Linear sweep voltammetry (LSV) curves for the oxygen reduction reaction (ORR) over Co-14MR/C-T catalysts (a) before and (b) after 50 potential-sweep cycles and for CoPc/C-T catalysts (c) before and (d) after 50 potential-sweep cycles between 1.0 and 0 V.

Table S1. The Fe loadings, densities of active Fe sites and TOF values for the data previously reported for Fe-N-C catalysts.

Catalyst	Fe loading[a] / wt%	Mass activity[b] / A g^{-1}	Active Fe site density[c] / $\times 10^{19}$ site g^{-1}	TOF[b] / electrons $site^{-1}$ s^{-1}	Ref.
CNRS	2.5	1.5	1.44	0.65	35
ICL	1.0	1.3	0.86	0.96	35
PAJ	0.6	2.9	0.25	7.23	35
UNM	0.8	3.5	0.63	3.45	35

[a] Determined by inductively coupled plasma atomic emission spectroscopy. [b] Evaluated from the current density at 0.8 V vs. RHE. [c] Determined by a NO_2^- reduction method.

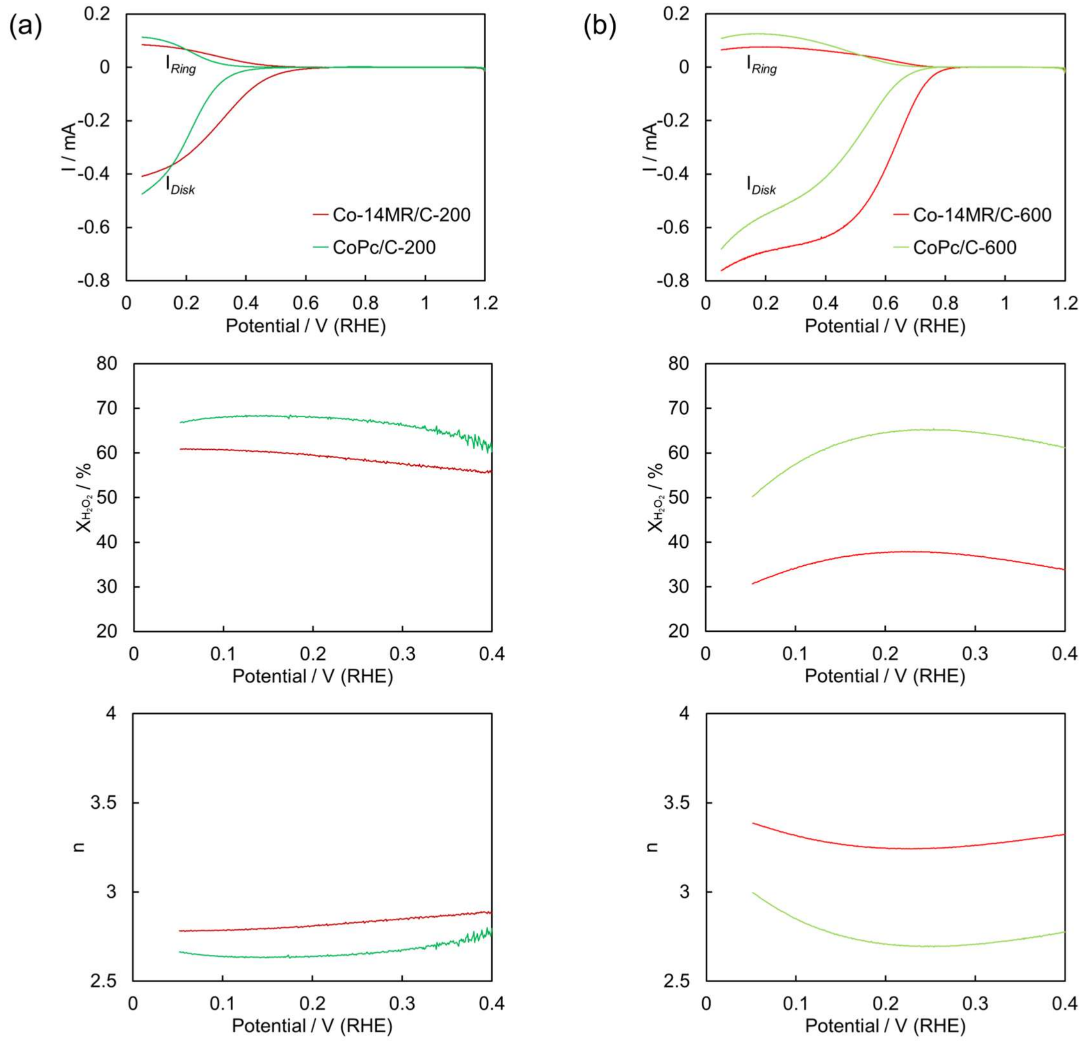

Figure S2. Rotating ring disk electrode polarization curves, H_2O_2 formation rates, and numbers of electrons involved in reactions over (a) Co-14MR/C-200 and CoPc/C-200 catalysts and (b) Co-14MR/C-600 and CoPc/C-600 catalysts in O_2-saturated 0.5 M H_2SO_4 solutions acquired at a sweep rate of 10 mV s^{-1} and rotation rate of 1600 rpm.

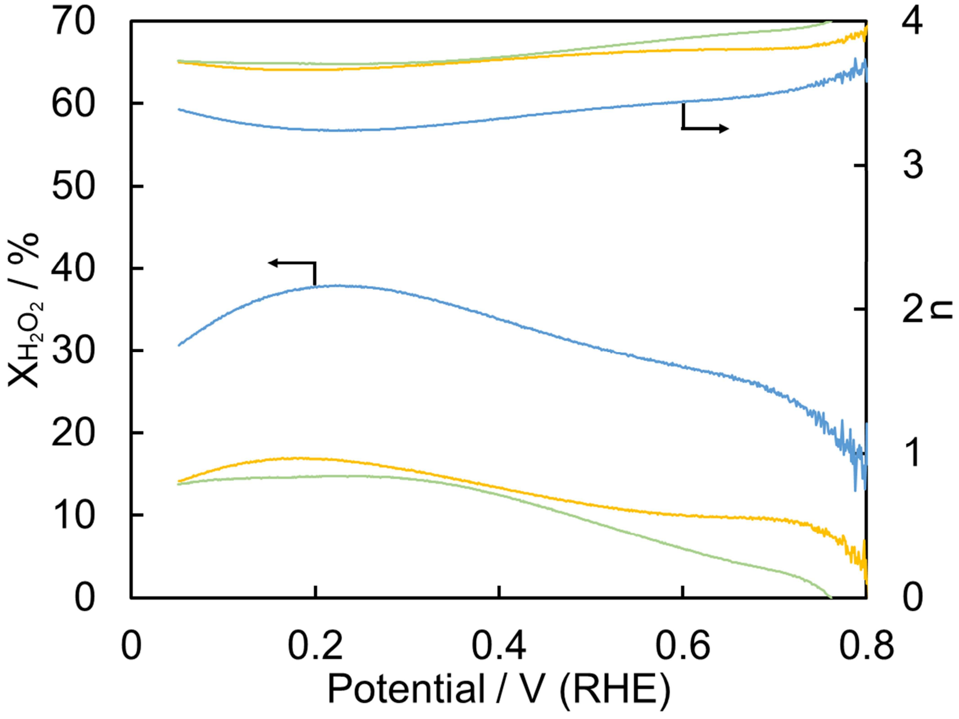

Figure S3. H_2O_2 formation rates and reaction electron numbers for the ORR on the Co-14MR/C-600 catalyst with 60 μg_{cat} cm^{-2} (blue), 200 μg_{cat} cm^{-2} (orange), and 400 μg_{cat} cm^{-2} (green) in O_2-saturated 0.5 M H_2SO_4 solutions acquired at a sweep rate of 10 mV s^{-1} and rotation rate of 1600 rpm.

Table S2. Demetallation ratios of Co-14MR/C-200, Co-14MR/C-600, Fe-14MR/C-200, and Fe-14MR/C-600 after 125 and 1200 cyclic voltammetry (CV) cycles between 1.0 and 0 V.

	CV cycle number	Demetallation ratio / %
Co-14MR/C-200	125	0.2(0)
	1200	1.1(1)
Co-14MR/C-600	125	5.6(1)
	1200	5.9(1)
Fe-14MR/C-200	125	56.9(69)
	1200	94.3(52)
Fe-14MR/C-600	125	29.6(68)
	1200	40.0(111)

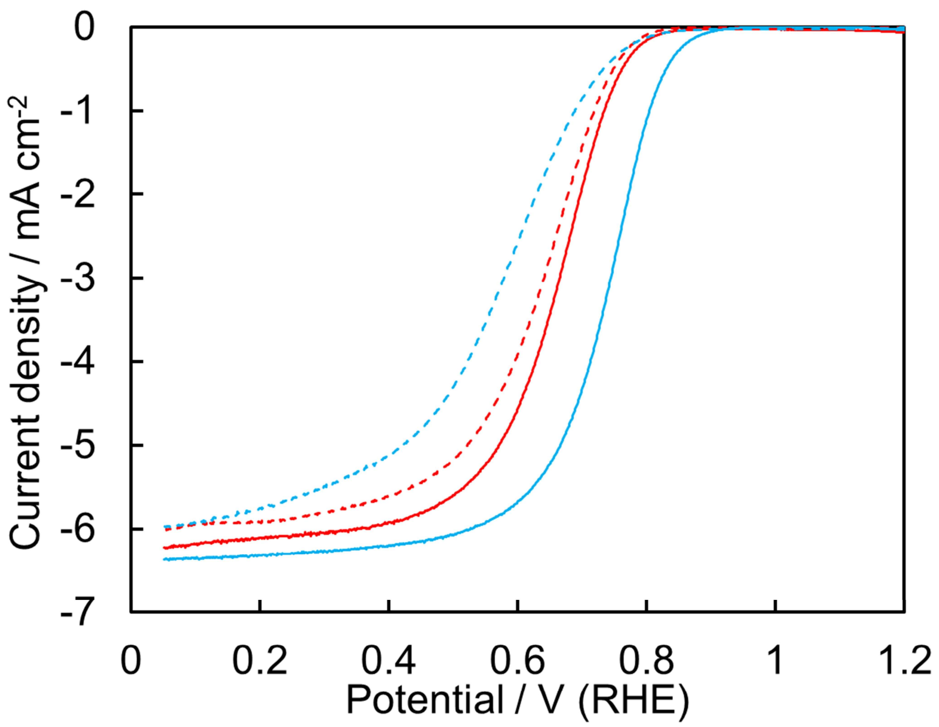

Figure S4. LSV curves for the ORR over Co-14MR/C-600 (red) and Fe-14MR/C-600 (blue) catalysts before (solid line) and after 1200 (dashed line) potential-sweep cycles between 1.0 and 0 V.

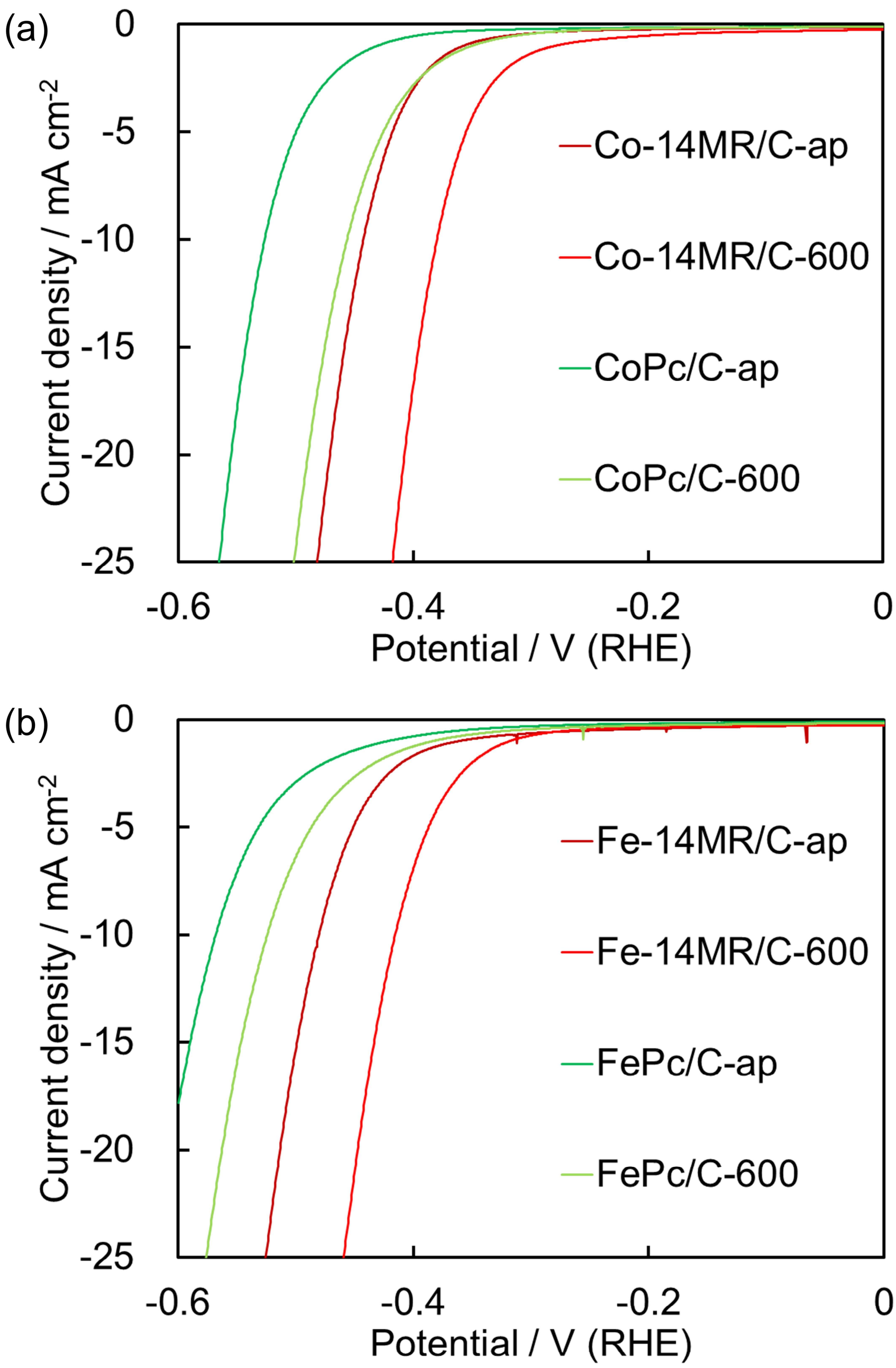

Figure S5. Initial LSV curves for the hydrogen evolution reaction (HER) over (a) Co-14MR/C-ap, Co-14MR/C-600, CoPc/C-ap, and CoPc/C-600 and (b) Fe-14MR/C-ap, Fe-14MR/C-600, FePc/C-ap, and FePc/C-600.

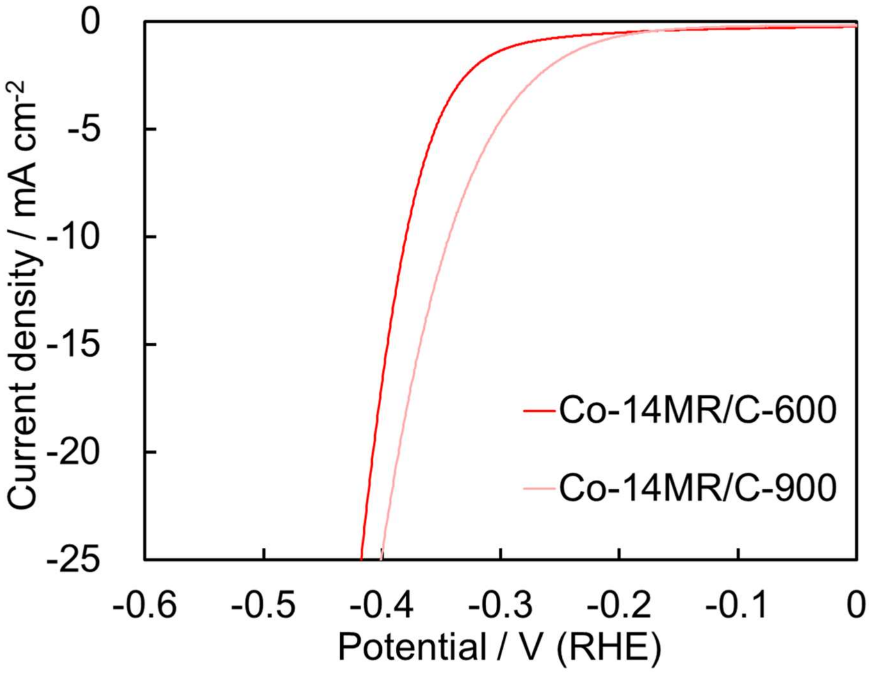

Figure S6. Initial LSV curves for the HER over Co-14MR/C-600 and Co-14MR/C-900.

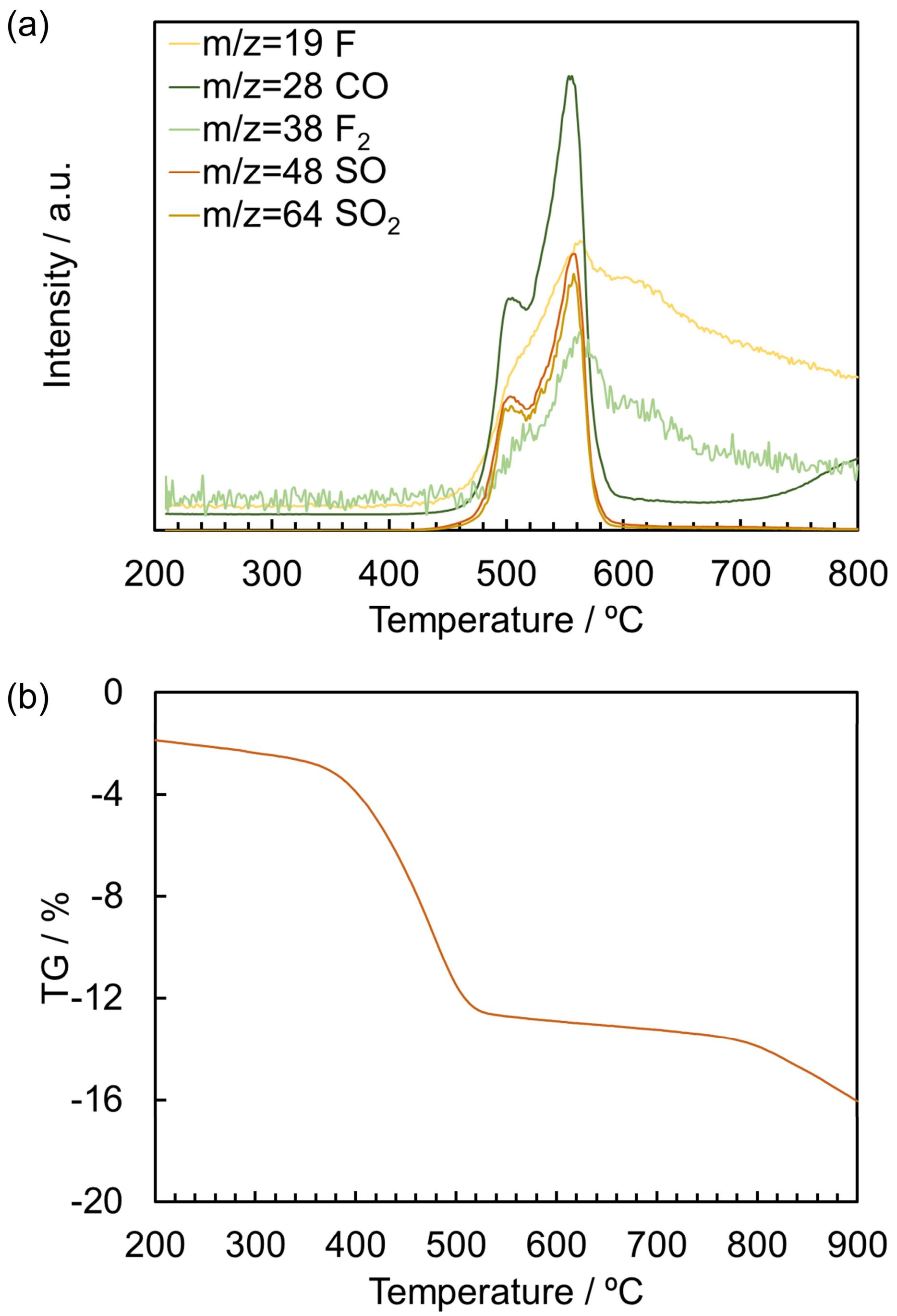

Figure S7. (a) Temperature programmed desorption (TPD) profile of Co-14MR/C: *m*/*z* = 19 (F), 28 (CO), 38 (F_2), 48 (SO), and 64 (SO_2). (b) Thermogravimetry analysis (TGA) curve for Co-14MR/C.

Table S3. The atomic ratio on surface (by XPS) of the Co-14MR/C-T catalysts.

	C / %	Co / %	N / %	N/Co
Co-14MR/C-ap	98.05	0.30	1.65	5.6
Co-14MR/C-200	97.59	0.34	2.07	6.1
Co-14MR/C-300	97.76	0.33	1.91	5.8
Co-14MR/C-400	97.97	0.30	1.73	5.8
Co-14MR/C-500	98.04	0.31	1.66	5.5
Co-14MR/C-600	97.54	0.37	2.09	5.8
Co-14MR/C-700	99.01	0.17	0.82	4.9
Co-14MR/C-800	99.12	0.15	0.73	4.8
Co-14MR/C-900	99.06	0.18	0.76	4.3
Co-14MR	83.22	2.49	14.29	5.9

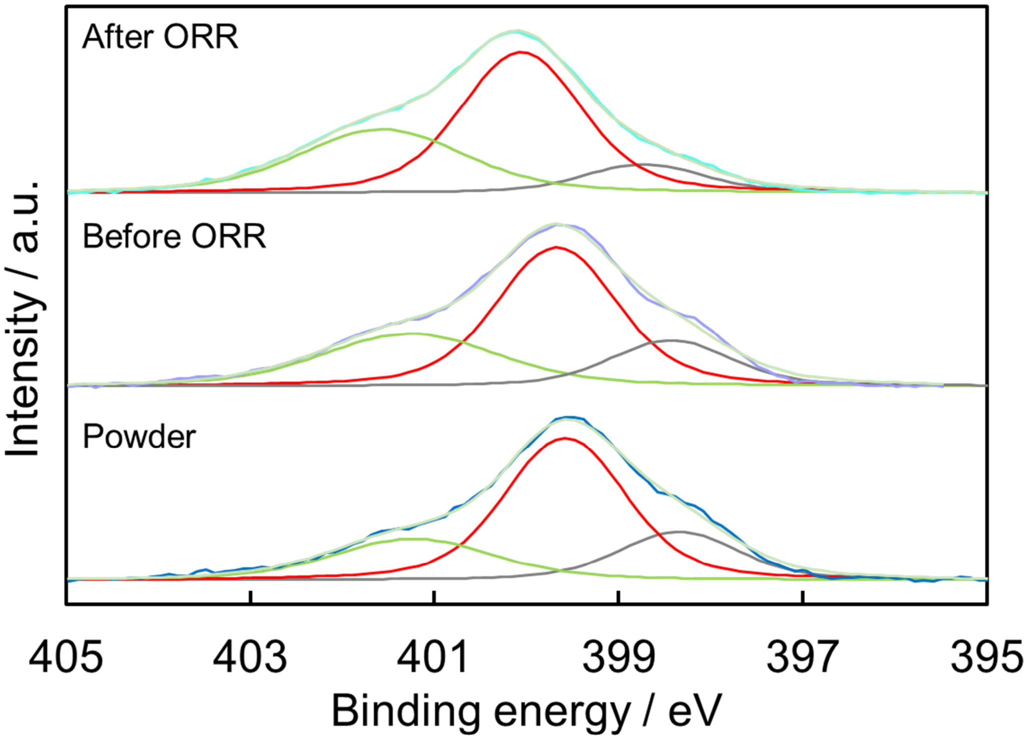

Figure S8. N 1s XPS of Co-14MR/C-600 on a glassy carbon electrode before and after ORR, together with that in powder form.

Table S4. N1s XPS analysis results of Co-14MR/C-600 on a glassy carbon electrode before and after ORR, together with that in powder form.

	B.E. / eV	Functional group	Atomic / %	FWHM / eV
Powder	398.35	Neutral Imine	20	1.55
	399.58	Co-N	58	1.55
	401.23	N-oxide/Quaternary N	22	2.09
Before ORR	398.43	Neutral Imine	18	1.55
	399.67	Co-N	53	1.55
	401.26	N-oxide/Quaternary N	29	2.26
After ORR	398.72	Neutral Imine	11	1.55
	400.06	Co-N	55	1.55
	401.57	N-oxide/Quaternary N/Protonated imine	34	2.12

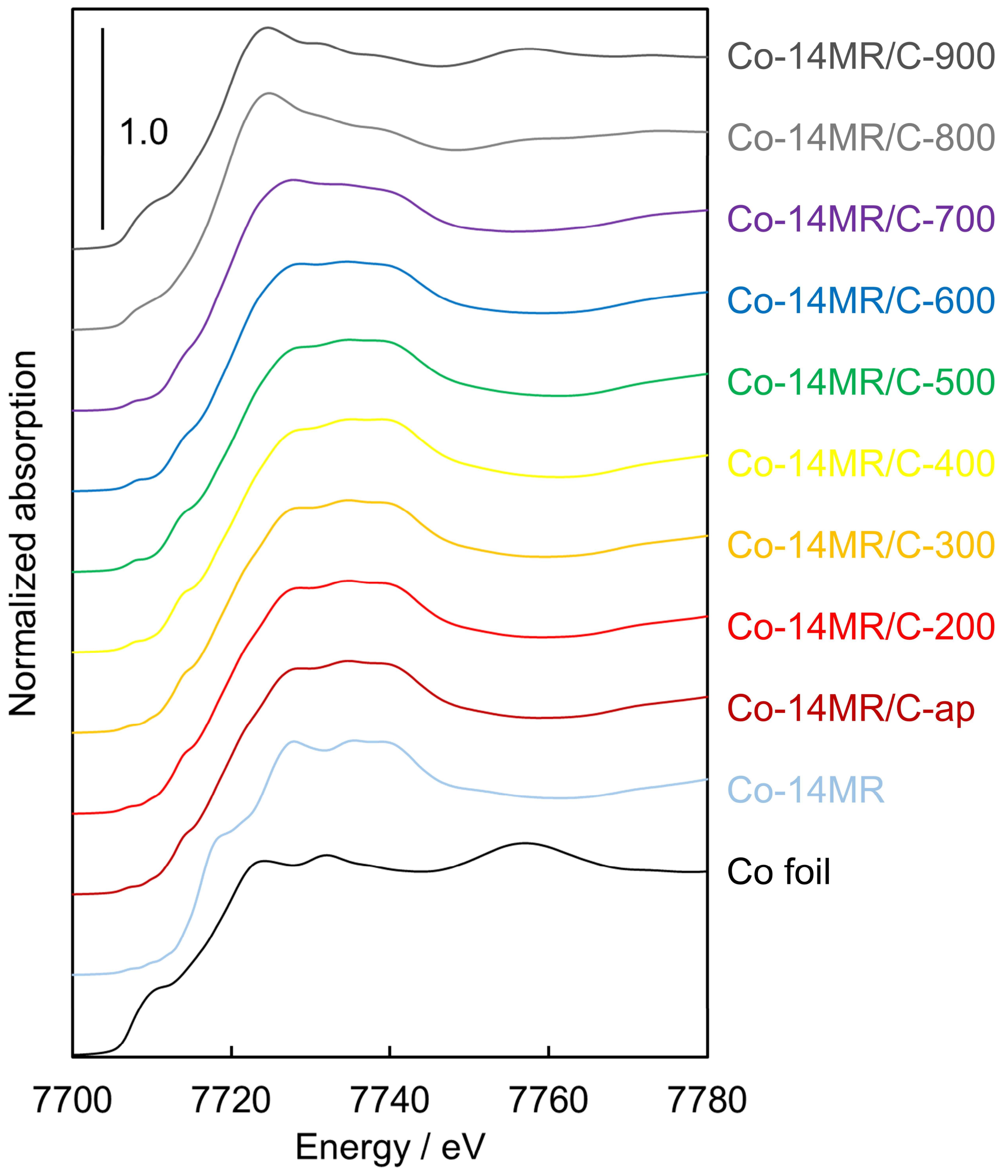

Figure S9. Co K-edge XANES spectra of Co-14MR/C-T catalysts, Co-14MR complex (amine form), and Co foil.

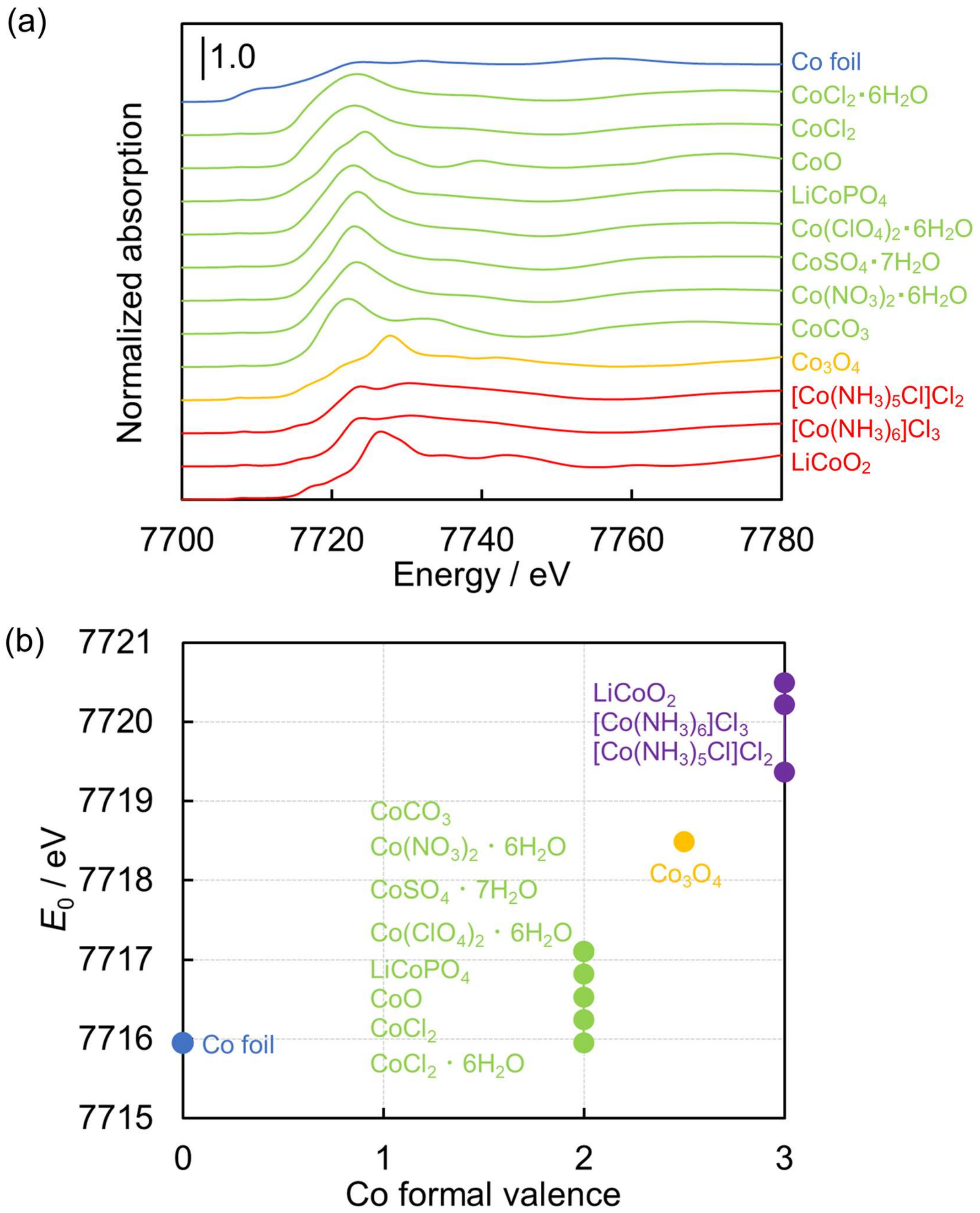

Figure S10. (a) Co K-edge X-ray absorption fine structure (XAFS) spectra of reference materials including Co foil, $CoCl_2{\cdot}6H_2O$, $CoCl_2$, CoO, $LiCoPO_4$, $Co(ClO_4)_2{\cdot}6H_2O$, $CoSO_4{\cdot}7H_2O$, $Co(NO_3)_2{\cdot}6H_2O$, $CoCO_3$, Co_3O_4, $[Co(NH_3)_5Cl]Cl_2$, $[Co(NH_3)_6]Cl_3$, and $LiCoO_2$. (b) Absorption edge energy at a normalized absorbance of 0.5 as a function of Co formal valence of reference materials.

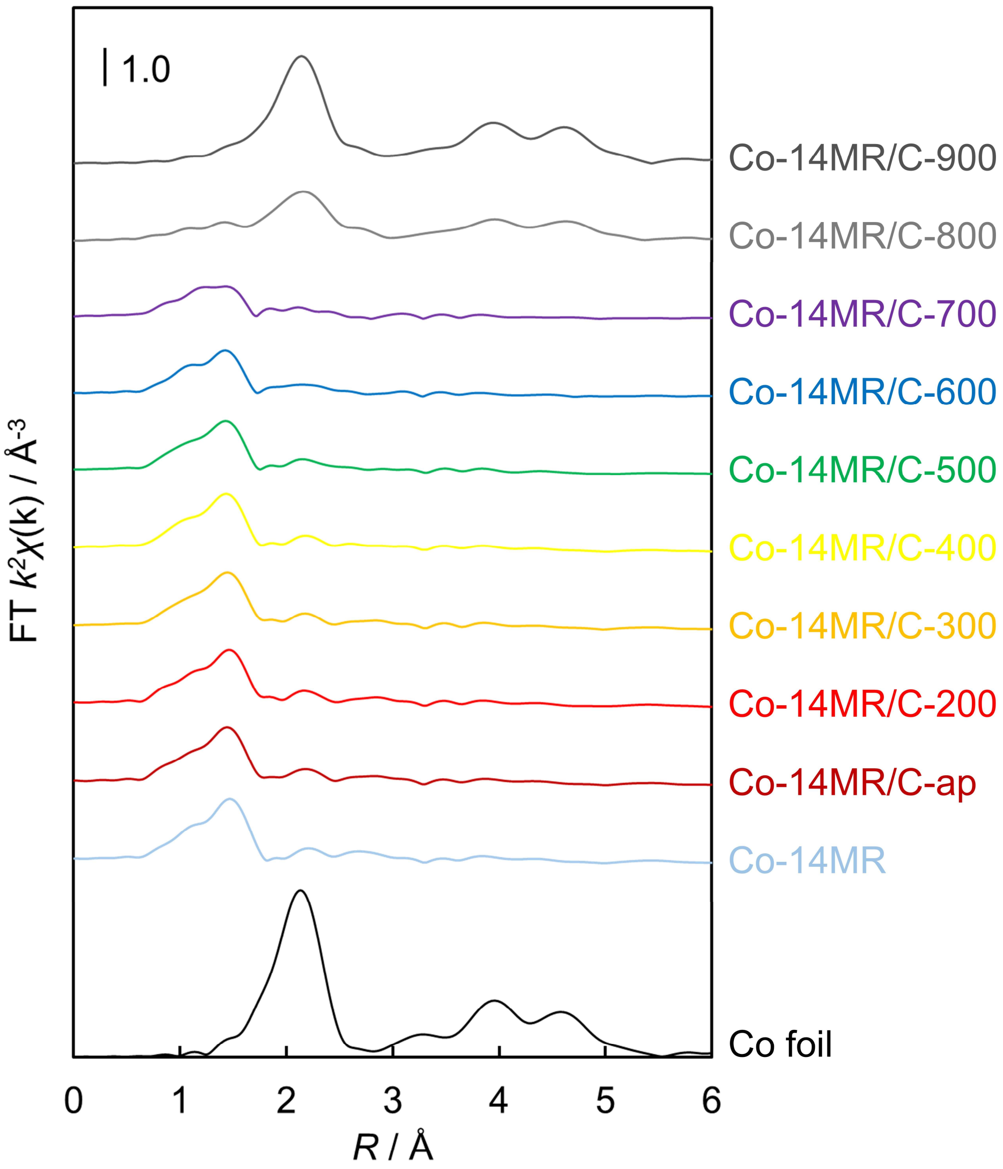

Figure S11. Co K-edge FT EXAFS spectra of Co-14MR/C-T catalysts, Co-14MR complex (amine form), and Co foil.

Table S5. EXAFS curve-fitting results for Co-14MR/C-T (T ≤600 ºC).[a]

T / ºC	CN[b]	R[c] / Å	σ^2[d] / Å^2	E_0[e]	R-factor
200	3.1(10)	1.86(2)	0.001(3)	7718(5)	0.036
300	3.2(11)	1.86(2)	0.002(3)	7718(6)	0.041
400	3.4(10)	1.85(2)	0.002(2)	7716(5)	0.026
500	3.3(9)	1.85(2)	0.002(2)	7715(5)	0.022
600	2.9(9)	1.85(2)	0.003(2)	7714(6)	0.026

[a] FT range: 3–14 Å^{-1}, curve-fitting range: 1.0–2.0 Å. [b] Coordination number. [c] Atomic distance. [d] Debye–Waller factor. [e] Absorption edge energy.

Because of the almost indistinguishable backscattering amplitudes and phase shifts of Co-N and Co-O scattering, the first shell was assumed to correspond to Co-N scattering. There was no significant difference in bond length between the samples. In addition, the coordination number (CN) remained approximately 3. These results suggest that the Co-14MR structure is stable at ≤600 °C.

Table S6. EXAFS curve-fitting results for Co-14MR/C-600, CoPc/C-600, Fe-14MR/C-600, and FePc/C-600.[a]

Catalyst	CN[b]	R[c] / Å	σ^{2}[d] / Å^2	E_0[e]	R-factor
Co-14MR/C-600	2.9(9)	1.85(2)	0.003(2)	7714(6)	0.026
CoPc/C-600	3.5(13)	1.90(3)	0.004(3)	7717(6)	0.038
Fe-14MR/C-600	5.9(15)	1.91(3)	0.015(3)	7111(4)	0.006
FePc/C-600	6.6(5)	1.98(1)	0.013(1)	7115(1)	0.001

[a] FT range: 3–14 Å^{-1}, curve-fitting range: 1.0–2.0 Å. [b] Coordination number. [c] Atomic distance. [d] Debye–Waller factor. [e] Absorption edge energy.

Table S7. Calculated adsorption free energies for oxygen species during the ORR and limiting potentials of Model A, Model B, and CoPc based on the computational hydrogen electrode model.

Model	Free energy at 300 K / eV				Limiting potential / V
	ΔG_{O2}	ΔG_{OOH}	ΔG_{O}	ΔG_{OH}	
A	4.92	4.73	3.43	1.41	0.19
B	4.92	4.56	3.00	1.26	0.36
CoPc	4.92	5.01	3.65	1.73	-0.09
